# Neural basis for categorical boundaries in the primate pre-SMA during relative categorization of time intervals

**DOI:** 10.1038/s41467-018-03482-8

**Published:** 2018-03-15

**Authors:** Germán Mendoza, Juan Carlos Méndez, Oswaldo Pérez, Luis Prado, Hugo Merchant

**Affiliations:** 1 Instituto de Neurobiología, UNAM, Campus Juriquilla, Queretaro, 76230 Mexico; 2 Department of Physiology, Anatomy and Genetics, University of Oxford, Oxford, OX1 3PT UK

## Abstract

Perceptual categorization depends on the assignment of different stimuli to specific groups based, in principle, on the notion of flexible categorical boundaries. To determine the neural basis of categorical boundaries, we record the activity of pre-SMA neurons of monkeys executing an interval categorization task in which the limit between short and long categories changes between blocks of trials within a session. A large population of cells encodes this boundary by reaching a constant peak of activity close to the corresponding subjective limit. Notably, the time at which this peak is reached changes according to the categorical boundary of the current block, predicting the monkeys’ categorical decision on a trial-by-trial basis. In addition, pre-SMA cells also represent the category selected by the monkeys and the outcome of the decision. These results suggest that the pre-SMA adaptively encodes subjective duration boundaries between short and long durations and contains crucial neural information to categorize intervals and evaluate the outcome of such perceptual decisions.

## Abstract (web summary)

Grouping stimuli into categories often depends on a subjective determination of category boundaries. Here the authors report a neuronal population in pre-supplementary motor area whose peak activity predicts the categorical decision boundary between long and short time intervals on a trial-by-trial basis.

## Introduction

Categorization aids in giving meaning to objects or events in the environment by assigning them to separate classes or groups^1^. For instance, animals can be classified as prey or predators, and fruits as edible or poisonous. Abstract magnitudes such as the passage of time can also be categorized. Thus, the duration of events or the interval between stimuli can be classified as short or long^2–4^. Categories are often separated by boundaries and stimuli are treated as equivalent if they belong to the same category or as different if they belong to a different category even if they resemble each other^5^. In addition, categorization is flexible, new arbitrary categories can be learned, and the same object can be classified as belonging to different groups depending on where the subjective limit between categories are located^2,6–9^. Therefore, categorization is a core element of cognition and has an important role in the evolution of species, since it allows for the rapid selection of the proper behavioral responses across a wide range of stimuli^10^.

Recently, important discoveries have been made on the understating of the neural basis of categorization. Neurophysiologic studies in monkeys categorizing visual or somatosensory stimuli have shown that several cortical and subcortical areas represent the category membership of stimuli at the single-cell level (medial premotor cortex^11,12^; neostriatum^13–15^; lateral intraparietal area^7,16^; area 7a^9,17,18^; and prefrontal cortex^6,8,9,15,17,19^).

Several hypotheses have been proposed to explain how the brain assigns different stimuli to specific categories^1,20–23^. Most of them imply the comparison of the stimulus to be categorized against mental representations of the limit between potential categories and/or of their prototypes. Neural recording studies found that categorization is associated to a bottom-up transition from stimulus-related to category-related signals^9,11,24,25^. Recently, a cortical network model was developed to explain the categorization of the movement directions of a visual stimulus^23^. In this model, category-selective activity emerged in a layer of association neurons located between sensory and decision neurons through reward-dependent plasticity and was conditioned to the presence of choice correlated activity fluctuations on the association neurons^23^. Nevertheless, in these studies the category limits and/or prototypes remain implicit in the transformation from stimulus to category-selective responses and, in fact, the neural bases of these crucial category elements are still largely unknown.

Here we test whether a neural representation of categorical boundaries is implemented during a temporal categorization task, where Rhesus monkeys categorize the duration of eight intervals as short or long according to a criterion acquired immediately before performing the task^2,26^. Critically, in each recording session, we employ three independent blocks, each composed of eight intervals. Also, each block has a different between-categories boundary. Monkeys report their categorical decision by moving a cursor inside an orange circle for short durations and inside a blue one for long durations. From trial to trial, these two targets could appear in any of eight possible positions, precluding the animals’ anticipation of the direction of their movement. Consequently, this experimental design allowed us to search for the neural correlates of flexible categorical boundaries and category-selective responses without the contamination of neural activity related to motor planning.

We recorded single-cell activity in the pre-supplementary motor cortex (pre-SMA), as it is well known that this area is a major node in the time processing network^27–36^. The results show that some pre-SMA cells encode the boundary between categories by reaching a constant peak of activity close to the limit between the short and long intervals within a block of trials. Interestingly, a subgroup of these cells dynamically changes the moment at which they reach this peak depending on the location of the categorical boundary of the current block of trials. A trial-by-trial analysis reveals that monkeys solve the categorization task by comparing this categorical boundary representation with that of the interval. We provide a realistic neural-network model for this comparison. Furthermore, within the pre-SMA there are partially overlapping neural populations that represent the category selected by the monkeys and the outcome of the decision. Consequently, these results suggest that the pre-SMA adaptively encodes the subjective boundary between short and long durations and is also involved in the processing of the additional information needed to generate categorical decisions and to evaluate the corresponding outcome.

## Results

### Interval categorization task

**Fig. 1:**
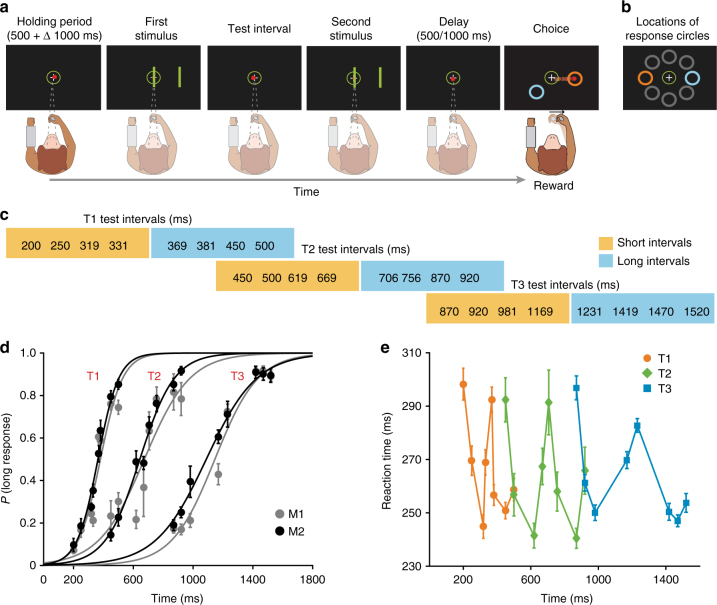
Trial events during the interval categorization task and behavioral results. **a** Monkeys categorized the interval between the first and second stimulus presentation as “short” or “long” by moving the red cursor into the orange or blue circular targets, respectively. **b** An example of a possible target configuration, with the response circles 180° apart. Gray circles (not visible in the actual task) show the eight possible target locations. **c** The three blocks of stimuli employed in this study (T1, T2, and T3). The short/long implicit limits for T1, T2, and T3 are 350, 685, and 1195 ms, respectively. Note that some time durations could be correctly categorized either as “short” or as “long” depending on the particular block presented to the monkey. **d** Psychometric curves for the two monkeys for the three blocks of durations. Dots represent the mean (±SEM) probability of categorizing a particular interval as long. **e** Mean (±SEM) of the reaction times of the monkey 1 as a function of the test intervals for the three blocks of stimuli

Monkeys categorized the interval between two visual stimuli as “short” or as “long” by selecting an orange or a blue target, respectively (Fig. 1a, Methods section). To start a trial, the animals gazed inside a fixation point and placed and maintained a cursor inside a central circle. Then, a brief visual stimulus (two parallel bars) was presented twice, with each presentation separated by a test interval. After a fixed delay, the two response targets were presented, and the monkey moved the cursor into the target that expressed his categorical decision (Fig. 1a, b). Within a trial, the response targets were placed in one of eight possible locations on the periphery of the fixation point (Fig. 1b). Crucially, on every recording session monkeys categorized three different blocks of stimuli (T1, T2, and T3), each containing eight different intervals. The shortest four intervals of every block were considered “short” and the remaining four, “long” (Fig. 1c). Moreover, depending on the particular block being categorized by the monkey, some duration could be correctly categorized either as “short” or as “long” (e.g., the 450 ms interval for T1 and T2). Thus, to categorize the stimuli, animals first had to acquire a categorization criterion. This happened during the initial trials (known as “training phase”), in which only the shortest and the longest intervals (the reference intervals) of that block were presented in an alternate fashion. The visual stimuli that delimited the intervals were orange for the short and blue for the long intervals. We expected that in this way, the monkeys would generate a mental implicit value that would serve as a limit or boundary between categories. Next, the animals entered the “testing phase” in which the stimuli were always green, and the eight intervals of the block were presented randomly.

### Behavior

**Fig. 2:**
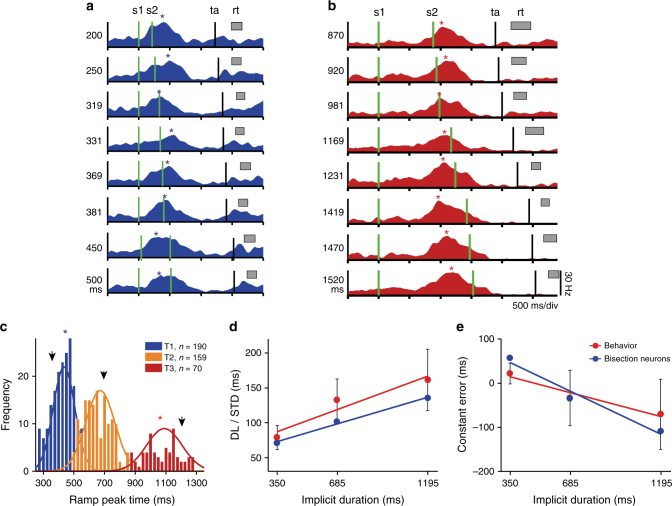
‘Boundary’ neurons encode the boundary between short and long categories across stimulus blocks. **a** Mean SDFs of a neuron for the test intervals of the T1 block. The two green vertical lines correspond to the two stimuli (s1 and s2) that define the test interval; the SDFs are aligned to the first stimulus. The time of peak activity (asterisk) occurs close to the implicit limit between short and long categories for this block (350 ms). The black vertical line indicates the target presentation (ta) and the gray rectangle the standard deviation of the movement initiation (rt). **b** Activity of the same neuron in **a** but recorded in the T3 block. The peak of activity shifted to the right, close to the limit for this block (1195 ms). **c** Histograms and corresponding Gaussian fittings of the mean times of peak activity for all boundary neurons across the three blocks (see the inset for color code). Arrowheads show the location of the implicit limit for each block. Asterisks indicate the mean times of peak activity of the neuron in **a**, **b**. **d** DL from both monkeys (red) and the standard deviation of the Gaussian functions in **c** as a function of the implicit limit for the three blocks. Lines show the best linear fits to the data. The linear increase in temporal variability (threshold) as a function of implicit limit duration (i.e., the scalar property of interval timing) is similar to the increase in the standard deviation of the peak time distributions for boundary neurons across the three blocks. The slope and constant are 54.5 and 0.09 for the psychometric behavior, and 45.2 and 0.06 for the boundary neuron distributions (both fits with *p* < 0.05). **e** Comparison between the behavioral constant error (mean ± SEM) and the time difference between the mean of the Gaussian functions in **c** and the implicit limit for the three blocks. Both the behavioral (red) and neural measures overestimate the shortest block (T1) and underestimate the longest block (T3)

The psychometric curves of both monkeys followed a typical sigmoid shape (Fig. 1d). For every block of stimuli, the probability of categorizing a particular interval as long increased as a function of the interval duration. Consequently, correct responses were more frequent for the shortest and longest intervals of a block, and errors were mostly observed for the intermediate intervals. In addition, we found that the slopes of the psychometric curves decreased as a function of the block of intervals (Fig. 1d), which indicates that the sensitivity of the monkeys diminished as a function of the duration of the intervals being categorized^2^. In fact, the difference limen (DL) increased linearly as a function of the block of intervals (Fig. 2d; red dots and line; Kruskal–Wallis test, H_2_ = 50.95, *p* = 8.62, *N* = 816; e−12; *r* = 0.71, *p* = 1.13 e−13; Table 1), following the scalar property of interval timing, which is a form of Weber’s law that has been widely documented across different motor and perceptual timing tasks^37,38^. Importantly, the wider time ranges employed in T2 and T3 could also contribute to the differences in slopes. The point of subjective equality (PSE), a measure of the subjective boundary between categories, was calculated from the psychometric curves as the interval at ‘*p* long’ = 0.5. The PSE can be directly compared with the actual category boundary by calculating the constant error (CE: the difference between the PSE and the implicit boundary between categories). In both monkeys, the CE was close to zero for the three blocks, which implied that the estimation of the implicit value was accurate. Nevertheless, the CE showed a small linear decrease as a function of the implicit category boundary (Fig. 2e; red dots and line; Kruskal–Wallis test, H_2_ = 22.71, *p* = 1.17 e−5, *N* = 816; *r* = −0.51, *p* = 1.18 e−6; Table 1). This last observation indicated that monkeys slightly underestimated the block T1 and overestimated the block T3. This corresponds to a slight overestimation of the boundary between short and long categories in T1 and to a boundary underestimation in T3. Similar biases in temporal estimation across blocks with different intervals have been consistently reported in the human timing literature^39,40^ further validating the monkey as a good animal model to study time categorization.

**Table 1:** Psychophysical measurements

	Monkey 1			Monkey 2		
	T1	T2	T3	T1	T2	T3
Implicit category boundary	350	685	1195	350	685	1195
PSE mean (SEM)	381.4 (7.11)	682.9 (45.22)	1146.4 (17.55)	361.6 (4.42)	643.9 (12.59)	1079.1 (17.88)
DL mean (SEM)	82.57 (4.43)	156.48 (17.62)	156.93 (9.97)	77.78 (5.26)	123.14 (6.14)	181.2 (7.11)
CE mean (SEM)	31.41 (7.11)	−2.13 (45.22)	−48.63 (17.55)	11.55 (4.42)	−41.14 (12.59)	−115.88 (17.88)

We also observed that intervals near the category boundary, which were harder to categorize, were associated with an increase in reaction time (RT)^41^ (Fig. 1e, Supplementary Fig. 1). We performed an ANOVA, using the RT as dependent variable and the ordinal interval (1–8) and the block number (T1, T2, and T3) as factors. The results showed significant main effects for ordinal magnitude [monkey 1: two-way ANOVA, *F*(7,320) = 33.21, *p* < 0.001, *N* = 344; monkey 2: *F*(7,257) = 9.89, *p *< 0.001, *N* = 280], but not for block [monkey 1: *F*(2,320) = 1.3, *p* = 0.272; monkey 2: *F*(2,256) = 2.31, *p* = 0.101] or the ordinal *x*-block interaction [monkey 1: *F*(14,320) = 0.61, *p* = 0.85; monkey 2: *F*(14,256) = 0.95, *p* = 0.50], supporting the view that it was the proximity of an interval to the boundary, which increased the difficulty for categorizing it, not its absolute duration.

Altogether, these behavioral results indicate that the monkeys were able to extract the temporal information of visually defined intervals in order to categorize their duration on the basis of previously instructed short and long prototypes. Additionally, the psychometric analysis demonstrated that monkeys were also successful at changing their decision criterion in order to correctly categorize the test intervals belonging to the different blocks of stimuli within the recording sessions.

### Neurophysiology

A total of 259 neurons were bilaterally recorded from the pre-SMA of monkey 1 and 723 neurons from the right pre-SMA of monkey 2 during the performance of the interval categorization task (Supplementary Fig. 2a, b). We assessed single-unit stability across the different blocks of the task by measuring the similarity of the average spike waveforms and the inter-spike interval histograms (ISIHs)^32,42^. Based on this analysis, a total of 816 cells were considered stable for at least two consecutive blocks (196 from monkey 1 and 620 from monkey 2) and were studied further.

### pre-SMA neurons represent the boundary between categories

Within a recording session, monkeys had to change their decision criterion in order to correctly categorize the test intervals of different blocks of stimuli. To do this, one possibility is that monkeys calculated the limit between categories for every block of intervals and employed it as a decision rule for solving the tasks. Interestingly, we found a group of pre-SMA neurons whose activity resembled the subjective boundary between categories for every stimulus block. The instantaneous firing rate of these neurons steadily increased to reach a peak and posteriorly decreased back to the spontaneous activity levels during the course of a trial (Fig. 2a). This activity peak was reached at a relatively constant moment after the presentation of the first stimulus (i.e., the beginning of the test interval) for all the intervals of a block. Interestingly, for short test intervals this peak tended to occur after the presentation of the second stimulus, but for long intervals it occurred before it (Fig. 2a, b). Moreover, once the monkey switched to categorize the intervals from a different block, the mean peak time of these neurons was shifted accordingly, closely following the location of the theoretical implicit boundary (Fig 2b) . Therefore, we call these neurons “boundary neurons”. Figure 2a, b illustrates a neuron that showed a mean peak time in the T1 block at 441 ± 27 ms (mean ± SEM) after the beginning of the test interval, a value close to the implicit boundary of this block (350 ms; Fig. 2a). In contrast, during the block T3 the activity of the same neuron reached its peak at 1067 ± 25 ms, which is now closer to the implicit boundary of this block (1195 ms; Fig. 2b). Consequently, within a recording session, this neuron adapted its activity pattern to reach its peak at a moment that was close to the current implicit boundary between categories of a particular block of stimuli.

**Fig. 3:**
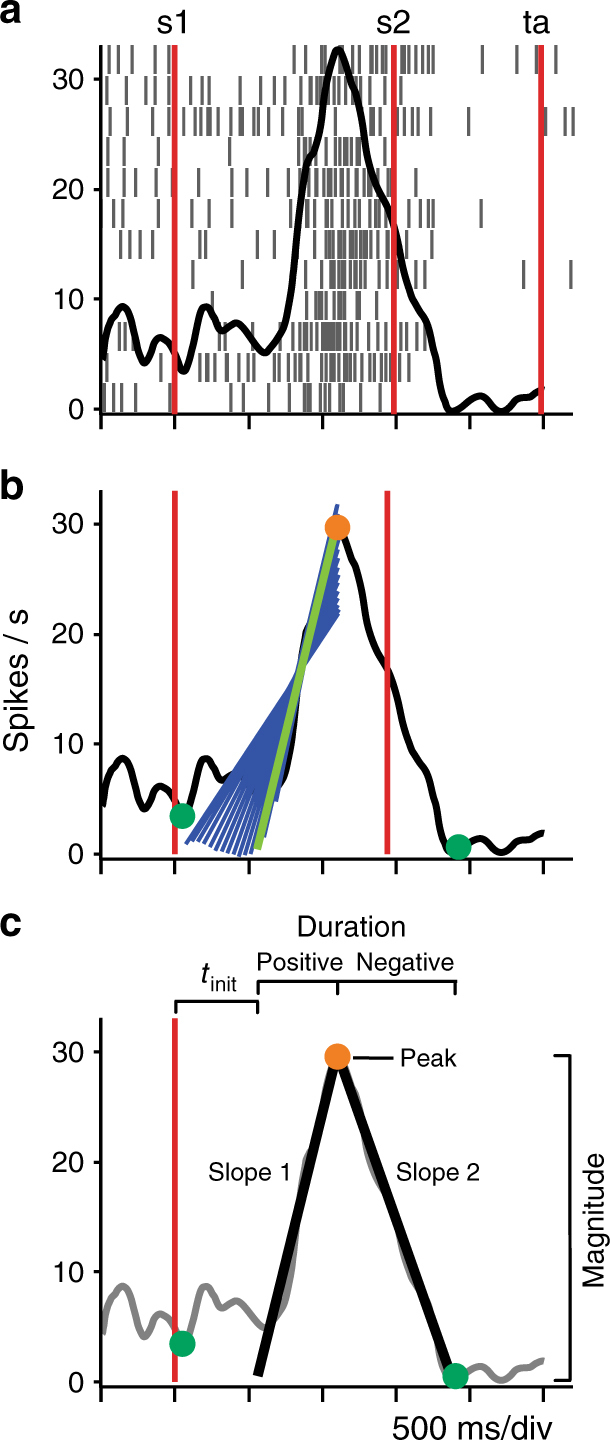
Iterative algorithm used to find the best regression model to describe the increase or decrease of instantaneous activity of boundary neurons over time with respect to a sensory event. **a** Raster plot and mean spikes density function (SDF; *σ* = 30 ms; black function) of the boundary cell in Fig. 2, for block 3 and the test interval duration of 1470 ms, aligned to the presentation of the first stimulus (s1). **b** A series of linear regression functions are displayed (blue lines), including the best model (thicker green line) identified by the algorithm. **c** Parameters extracted from the linear regression model for the identification of bisection neurons

The up–down activation profile of the boundary cells was characterized by the duration of the positive and negative consecutive ramps, the magnitude of the peak, and the time from the first stimulus to the activity peak using a linear regression iterative algorithm (Fig. 3a–c; Methods section). Thus, units were considered boundary cells when they met the following criteria: (1) They presented a significant up–down pattern of activity in at least 5 of the 8 test intervals in a block; (2) their peak activity was above 8 Hz on each activation period; (3) the time of peak activity was not statistically different across test intervals (Fig. 3, Methods section). Indeed, we performed an ANOVA using the peak time of boundary cells that were active on at least two blocks as dependent variable and the results showed significant main effects for block number (two-way ANOVA, *F*(2,1254) = 1336.6, *p* < 0.00001, *N* = 1255), but not for ordinal interval (*F*(7, 1254) = 1.82, *p* = 0.081) nor for the ordinal *x*-block interaction (*F*(14,1254) = 1.4, *p* = 0.145; Supplementary Fig. 3a). In addition, the duration of the up–down cycle of these cells increased as a function of block number (One way ANOVA, *F*(2,416) = 93.7, *p* < 0.00001, *N* = 417; Supplementary Fig. 3b). Considering both monkeys, 190, 159, and 70 neurons showed a boundary pattern of activity during T1, T2, and T3, respectively. Most of these cells encoded the boundary in only one block (*χ*^2^-test of independence, *x*^2^(1, *N* = 615) = 55.64; *p* = 8.66 e−14; see Methods section and Table 2); namely, different neurons encoded the boundary in different blocks. Nevertheless, we found 87 neurons, corresponding to 27% of the total boundary cell population, which showed a boundary pattern of activity on two or more blocks of stimuli (Table 2). Supplementary Fig. 4 compares the time to peak activity for the group of boundary cells recorded during only one block of stimuli with that recorded in two or three. It can be appreciated that the distribution of peak activity times for both groups is very similar; in fact, a Kruskal–Wallis test and Dunn-Sidak post hoc showed no significant differences between them, and only showed significant differences between the time to peak across blocks (Kruskal–Wallis test and Dunn-Sidak post hoc, *χ*^2^(5) = 312.62, *p* < 0.001, *N*_T1oneblock_ = 116, *N*_T2oneblock_ = 87, *N*_T3oneblock_ = 32, *N*_T1twoormore_ = 74, *N*_T2twoormore_ = 72, *N*_T3twoormore_ = 38; Supplementary Fig. 4). Moreover, 64.4% of the boundary neurons recorded in two or more blocks showed a significant shift of the time to peak in at least one pair of blocks (Wilcoxon signed-rank test or Kruskal–Wallis test and Dunn-Sidak post hoc test; *p* < 0.05).

**Table 2:** Total number of boundary neurons per task combination

Tasks	Number (%) of neurons
T1 T2 T3	10 (3.10)
T1 T2	49 (15.22)
T1 T3	15 (4.66)
T2 T3	13 (4.04)
T1	116 (36.02)
T2	87 (27.02)
T3	32 (9.94)

Next, we searched for correlations between the psychometric performance of monkeys and the equivalent measures from the population of boundary neurons. The relation between the subjective and the actual implicit boundary is captured by the CE. Positive CE values indicate overestimation of the actual boundary, which is equivalent to an underestimation of the durations in a block. To obtain an equivalent measure of CE for the population of boundary neurons, we calculated the difference between the mean of the fitted distributions in Fig. 2c (435, 660.5, and 1066.4 for T1, T2, and T3, respectively) and the actual implicit boundary of the corresponding block of stimuli (350, 685, and 1195 for T1, T2, and T3, respectively). The neural CE tended to underestimate the shortest block of intervals (T1) and overestimate the longest block (T3), similar to the psychometric behavior of the animals (Fig. 2e; constant and slope for behavior: 51.4 and −0.11; constant and slope for neurons: 113.6 and −0.19; both linear fits with *p* < 0.05). In addition, there was a close correspondence between the monkeys’ DL and the equivalent from the population of boundary neurons (the dispersion of the distribution of mean peak times in Fig. 2c). Both the monkeys’ DL and the standard deviation of the peak time distributions increased with a similar slope as a function of the implicit limit duration (Fig. 2d; constant and slope for behavior: 54.5 and 0.09; constant and slope for neurons: 46.7 and 0.075; both linear fits with *p* < 0.05).

Since the boundary cells were detected using an analysis window including the categorized interval plus 500 ms after the second stimulus, the shift of peak activity across blocks could result from the different size of the analysis window. Hence, we conducted the same analysis using a constant window of 700 ms before and after the second stimulus across durations and blocks on the original boundary cells and also with a window of fixed size across test intervals (the longest test interval of each block plus 500 ms before and after) and aligned to the first stimulus. The results showed that the peak times of boundary cells were similar with the three detection methods, showing a high correlation when computed with a variable or a constant window (variable window across trials and blocks vs fixed window aligned to the second stimulus: Pearson *R* = 0.958, *p* < 0.00001, *N* = 356; variable window across trials and blocks vs fixed window across trials and aligned to the first stimulus: Pearson *R* = 0.968, *p* < 0.00001, *N* = 331; Supplementary Fig. 5a, b). Furthermore, the peak of activity of boundary cells detected with a constant analysis window showed the same increase in standard deviation of peak time distributions and similar properties on the neural CE across blocks (Supplementary Fig. 5c, d). Finally, for the cells that showed boundary activity on two consecutive blocks, we calculated their peak activity times for shared test intervals, namely, 450 and 550 ms for blocks 1 vs 2; and 870 and 920 ms for blocks 2 vs 3. The mean time to peak of these boundary neurons showed a significant increase between consecutive blocks (Supplementary Fig. 6a, b). Moreover, we found similar effects when the same analysis was performed on the subset of cells that showed boundary activity in only one block (Supplementary Fig. 6c, d), suggesting that pre-SMA boundary neurons shift their time to peak across blocks at the single-cell level and/or at the population level.

Overall, these findings support the notion that the activity of the boundary neurons found in the pre-SMA corresponds to a subjective representation of the boundary between categories that changes between blocks of stimuli. Accordingly, we propose that this neural activity could serve as a decision criterion for the categorization of time intervals in the millisecond range. In the next section, we test this idea explicitly.

### Decisions are predicted on every trial from boundary neurons

**Fig. 4:**
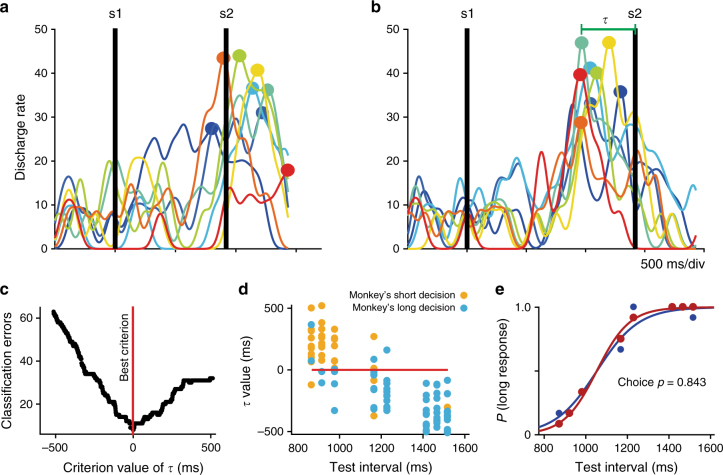
Trial-by-trial decoding of the monkey’s forthcoming perceptual decision employing the peak activity time of boundary neurons. **a** SDFs of the boundary neuron in Fig. 2a, b for trials in which the shortest test interval (870 ms, block 3) was presented to the monkey. Each color line represents an individual trial (eight trials are shown). Dots indicate the moment of the peak activity. s1 and s2: moments of presentation of the 1st and 2nd stimulus, respectively. Note that most activity peaks occur after the second stimulus. **b** Trial by trial activity of the same boundary neuron for one of the longer test intervals of the same block (1470 ms, block 3). Note that all activity peaks occur before the second stimulus. The interval between the peak activity (*τ*) and the second stimulus is indicated for one trial. **c** Number of errors in trial classification used as categorization cut criteria with a range from −500 to 500 ms in steps of 1 ms. The best limit criterion corresponds to the value of *τ* of the boundary neuron in **a**, **b** that minimized the classification error for trials as coming from actual short or long response trials. **d** Values of *τ* for the same cell as a function of the test intervals of the block 3 of the time categorization task. Note that for shorter intervals values were mainly positive, which implies that the peak activity occurred mainly after the presentation of the second stimulus. The opposite occurs for longer intervals. The red horizontal line corresponds to the best criterion in **c**. The color code is in the inset. **e** Psychometric (blue) and neurometric (red) functions. The neurometric curve was constructed from the trial probability below the best criterion as a function of test interval in **d**. Logistic functions were fitted to behavioral and neuronal data; the DLs were 112 ms for both curves and the PSE was 1052 ms for the behavior and 1105 ms for the neurometric function. Finally, the relation between the actual categorical behavior and the neural classification of the cell activity in **c** for the 96 trials was remarkably large (*χ*^2^-test = 63.8, *P* < 0.00001), as well as the mean boundary-choice probability (Methods section)

We hypothesized that the up–down pattern of activity of the boundary neurons can be employed by an “optimal neural decision reader” as a criterion to categorize intervals as short or long, depending on the temporal relation between these neurons’ activity peak and the occurrence of the second stimulus. To test this idea, we first evaluated whether the activity of single-boundary neurons could be employed on a trial-by-trial basis to predict the forthcoming categorical decision of the monkeys. We quantified the elapsed time (*τ*) between the peak of activity and the occurrence of the second stimulus for each trial of the eight test intervals (Fig. 4a, b) and then found the value (decoding criterion) that minimized the error to classify the stimuli as short or long short based on *τ* (Fig. 4c, d red line). Finally, a neurometric curve was constructed by computing the probability that the neuron chose a test interval as “short” or “long” based on the number of trials that were above or below the decoding criterion (Fig. 4d, e). A contingency table was calculated between the decoded and the observed monkey’s choices across all trials and a *χ*^2^-test was performed on the table. For the neuron in Fig. 4 the relation between psychometric and neurometric data were remarkably strong (96 trials; *χ*^2^-test = 63.8, *p* < 0.00001), supporting the notion that the activity of this boundary cell contributed to the monkey's choice. Consequently, the DL and PSE from the neurometric and psychometric functions were practically identical (neurometric DL: 111 ms, neurometric PSE: 1051 ms; psychometric DL: 112 ms, psychometric PSE: 1052 ms; Fig. 4e. See Supplementary Fig. 7 for more examples). We also computed an analog of the choice probability index^43^, which indicates the proportion of behavioral responses that can be predicted from the length of the neuron's *τ*. This index acquires values between 0 and 1 (where 1 indicates a perfect separation between the *τ* distributions for short and long responses), and was computed using only the neural and behavioral data of the intermediate intervals in a block, in order to obtain a balanced number of short and long categorical decisions for every interval^44^ (Methods section). The boundary-choice probability index of the neuron in Fig. 4 was 0.84. We found that 130 boundary neurons showed both a significant relation between neural decoding and behavioral performance (*χ*^2^-test, *p*< 0.05, 96 trials) and a boundary-choice probability index larger than 0.6.

In sum, these findings show that the relation between the peak activity of boundary neurons and the end of the test interval predicted, on a trial-by-trial basis, the forthcoming monkeys’ perceptual decision long before the actual response of the animal.

### Boundary neural population activity

**Fig. 5:**
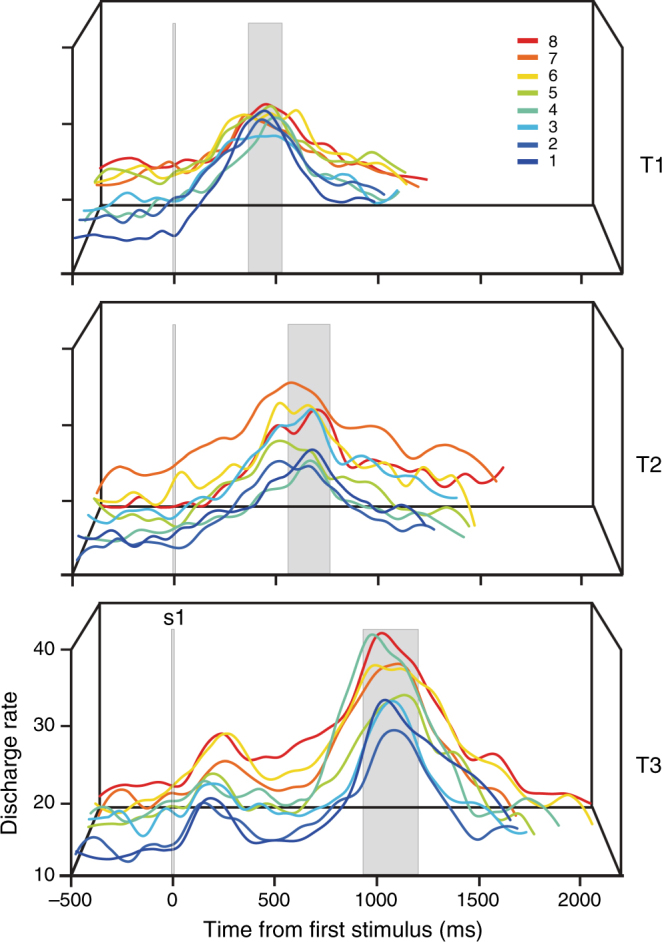
Mean activity of the population of boundary neurons for the three blocks of the categorization task (T1, T2, and T3). The color code corresponds to the eight test intervals as indicated in the inset. The vertical line indicates the presentation of the first stimulus (s1). The vertical rectangle indicates the mean (+1 SD) time to peak activity from first stimulus computed from the Gaussian distributions of Fig. 2c

Since the distribution of peak activity times in boundary neurons covers a range of values (Fig. 2c), we suggest a population code reads over time the activity of all boundary cells. Figure 5 shows the average population activity of boundary cells across the three blocks of stimuli. It is evident that the population response reached a peak within a range close to the implicit boundary across target intervals for the three blocks. Our hypothesis is that the population activity is the signal used by an optimal reader, which selects the short category when the neural response to the second stimulus occurs first, or the long category when the boundary cell population’s activity peak occurs first. This boundary population signal would depend on both the group of neurons that represent the boundary across blocks and the neurons that are recruited to encode the boundary for only one block of stimuli. In the next section, we show a model we developed to test how this could be implemented at the neural circuit level.

### A recurrent neural-network model of boundary cells

**Fig. 6:**
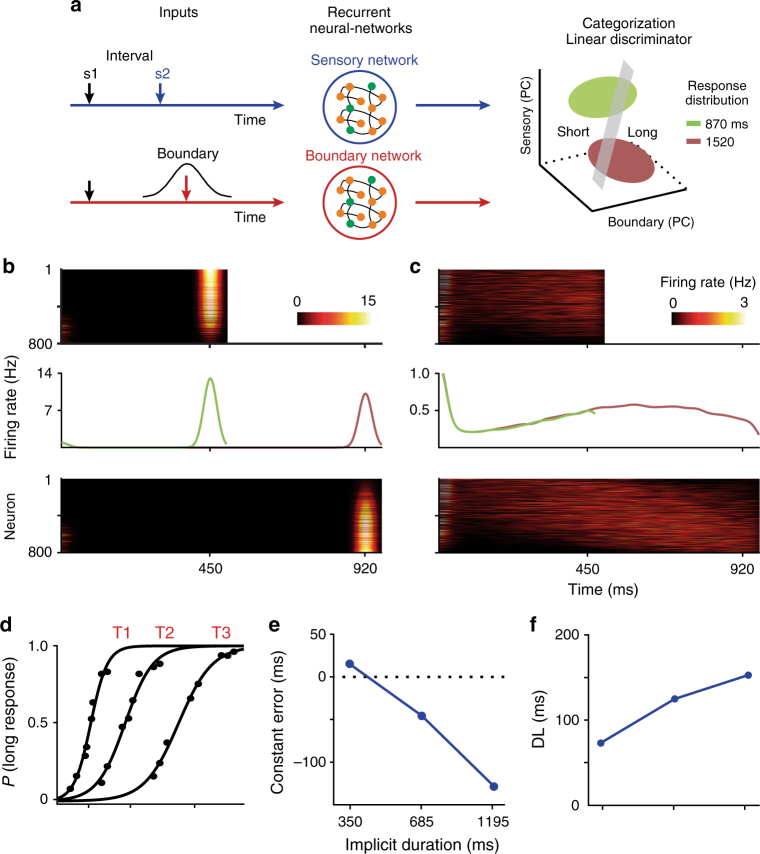
A neural-network model of boundary cells generating categorical signals. **a** Center panel, the model consisted of an independent sensory and a boundary recurrent network whose outputs were read by a linear classifier. Each network consisted of 800 excitatory and 200 inhibitory neurons. Left panel, the sensory network received an input comprising of two pulses (s1 and s2) separated by a test interval, whereas the boundary network received a set of pulses with intervals coming from a Gaussian distribution, simulating boundary input responses. The pulses modulated the activity of each network cell through two input current types: AMPA, which induced facilitation, and GABAb, which corresponded to a slow inhibitory current. Right panel, PCA was used to project the profile of neural activation of the sensory and boundary networks onto three dimensions (X = Boundary PC1, Y = Boundary PC2, Z = Sensory PC1). A linear classifier (gray plane) was used to find the plane in the PCA space that could divide short and long intervals according to the psychometric performance of the monkey. Thus, the proportion of simulations (green ellipse for shorter interval and brown ellipse for long interval) that was to the right of the discrimination plane was considered short, while the proportion to the left was considered long. **b** Temporal pattern of activity of the sensory network population during the presentation of a short and a long interval (upper and lower panels, respectively). After the presentation of the second pulse, at 450 or 920 ms, the network presented a large phasic activation. The mean population firing rate is indicated in the middle panel. **c** Temporal pattern of activity of the boundary network population during the presentation of the same short and long intervals of (**b**) (upper and lower panels, respectively). When the longest interval was presented, the population of cells in the boundary network generated an up–down profile of activation. **d** Networkmetric curves generated by the model. The probability of long categorization was computed from the linear classifier in **a** right. **e** Constant error and **f** DL produced by the model for the three blocks of stimuli employed in the time categorization task

As a proof of concept, we developed a neural-network model consisting of a linear classifier (Fig. 6a, right) that categorized interval durations as short or long based on the activity from two independent recurrent neural networks (Fig. 6a, center): one processing the information about the boundary between categories and another encoding the pair of visual stimuli delimiting the test intervals (Fig. 6a, left). The neurons in each network were modeled as Integrate-and-Fire cells with short- and long-term currents (Methods section). All neurons in these networks processed two stimuli separated in time by a particular duration: the neurons in the sensory network received two short pulses defining the test interval (Fig. 6b); in contrast, the inputs to the neurons in the boundary network were separated by Gaussian-distributed intervals, resulting in an up–down profile of activation with slightly different peaks for different neurons in this network (Fig. 6c). Overall, however, the population signal showed an activity peak that was close to the implicit interval, as observed on the actual neural population responses (Fig. 5). In addition, the Gaussian input distribution of the boundary network had a mean on the implicit interval of a block and a standard deviation that increased as a function of the blocks of stimuli simulating the properties of the boundary cells. A principal component analysis (PCA) was performed on the time-varying activity of the cells of the sensory and boundary networks to produce population state trajectories in three dimensions, including two for the boundary network (X: PC1, Y: PC2) and one for the sensory network (Z: PC1) (Fig. 6a, right; Supplementary Fig. 8a). The projection of the high-dimensional individual neural activity into a low-dimensional topological space can generate a robust and stable manifold that represents the latent task variable^45^. In this case, the trajectories reflect the occurrence of the stimuli, the categorical boundary representation, and the categorical decision over time. The network trajectories across trials showed a curvilinear path for the PC1 and PC2 of the boundary network for all intervals. In contrast, the PC1 of the sensory network showed a sharp upward shift for the second stimulus, which defined the end of the interval. A linear classifier was used to find the plane in the PCA space that could divide short and long intervals according to the psychometric performance of the monkey (Methods section). The classification plane (gray plane Fig. 6a right; Supplementary Fig. 8d) allowed us to compute the probability of classifying an interval as long based on the neural trajectories of 50 neural-network simulations (Supplementary Fig. 8d). Thus, the proportion of simulations lying to the left and right of the plane were considered short and long, respectively. This classifier also found the decision time in the trajectory where the neurometric performance was closest to the monkeys’ categorization behavior, corresponding to the moment where, theoretically, the decision was reached. For example, for the shortest test interval of block 3, the optimal decision time to classify the interval as short occurred just after the presentation of the second stimulus (Supplementary Fig. 8b), whereas for the longest test interval the optimal decision time of the classifier was before the second stimulus, close to the peak of the boundary network population activity (Supplementary Fig. 8c). The model generated neurometric curves (Fig. 6d) that showed a CE (Fig. 6e) and DL (Fig. 6f) that were very similar to the monkeys’ psychometric behavior across the three blocks (Figs. 1d, 2d, e). Therefore, the model’s predictions support the notion that an optimal reader uses the population signal of the boundary cells together with the detection of the second stimulus defining an interval to determine whether an interval is short or long, following the rule described in the previous section.

### Category-selective neurons

**Fig. 7:**
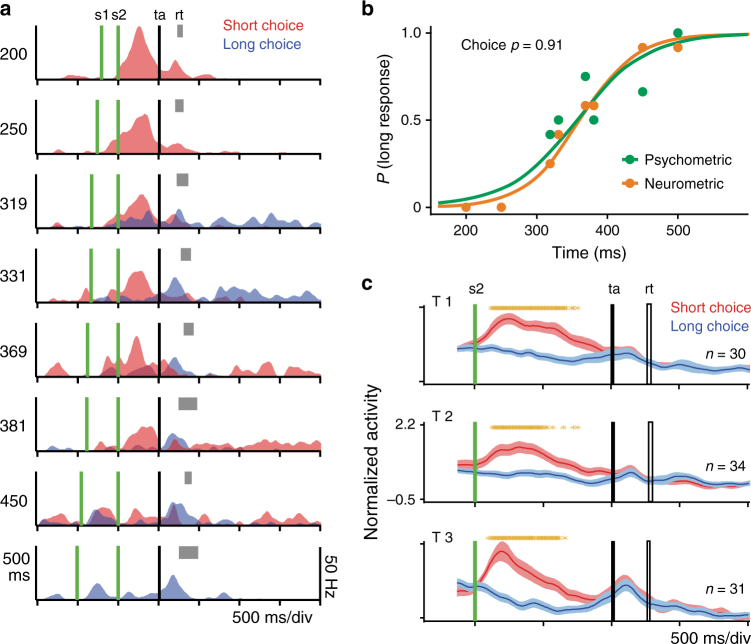
Category-selective neurons. **a** Mean SDFs of a category-selective neuron of monkey 2 whose activity was modulated during the delay epoch by the future monkey’s categorical choice for short durations. The activity is segregated by the monkey’s choice (red: short, blue: long choice) and interval duration (indicated to the left of each graph). Same notation as in Fig. 2a. **b** Psychometric performance (green) of the monkey during the recording of the neuron in **a**, whose neurometric function is shown in orange. The choice probability of this cell was close to 1 and the *χ*^2^-test on the contingency table calculated between the decoded and the observed monkey’s choices across all trials was significant (*x*^2^ = 54.43; *p* < 0. 001). **c** Population activity (mean ± SEM) of neurons of monkey 1 whose firing rate is significantly higher for short response trials during the delay epoch (red function). Orange asterisks indicate: (1) *t*-test significant differences in ten millisecond, non-overlapping bins (over the population SDF, short vs. long choices, 95% bootstrap confidence interval, 100 iterations). The single cell (**a**) and the population activity (**c**) are aligned to the presentation of the second stimulus (s2)

We also found a large group of pre-SMA cells that showed all the properties of category-selective neurons, that is, their activity differentiated between categories while showing a homogenous response within each category. Furthermore, their activity mirrored the categorization behavior of the monkeys, namely, both the neurometric and psychometric functions showed a sigmoidal shape with clear differences between categories and similar responses within each category. These neurons were identified using the following criteria. First, a multiple linear regression was performed with the discharge rate of a cell (in a sliding window of 250 ms in steps of 25 ms) as dependent variable, and the categorical choice of monkeys, the duration of the test stimulus, and the trial outcome (correct/incorrect) as factors. We performed this analysis during the delay period, the reaction and movement times, and during the intertrial period. Nevertheless, since most of the category-related activity showed a significant effect during the delay epoch (Supplementary Fig. 12), we focused our analysis of category-selective cells on this epoch. We calculated two additional measures that determined the association between the neural activity and the monkeys' choices for the same sliding windows. One is the choice-probability index^43^, which indicates the proportion of behavioral responses that can be predicted from the neuron's activity. In addition, a contingency table was calculated between the decoded and the observed monkey’s choices across all trials and a *χ*^2^-test was performed on this table (Methods section). A cell was considered category selective when: (1) the categorical choice factor was significant in the permutation test (*p* < 0.05) of the multiple linear regression model for at least two consecutive sliding windows during the delay period; (2) the choice probability index was above 0.6; and (3) the *χ*^2^-test was significant (*p* < 0.05). Figure 7a illustrates a category-selective neuron recorded during the T1 block. The activity of this neuron showed a significant increase during the delay epoch for trials selected as “short” by the animal. Furthermore, its choice probability index was 0.91, the *χ*^2^-test was *x*^2^ = 54.43 (*p* < 0.001, 96 trials), and the psychometric and neurometric functions had very similar sigmoidal profiles (Fig. 7b; neural PSE: 362.10; neural DL: 43.57; behavioral PSE: 356.75; behavioral DL: 55.22). We also found another group of category-selective neurons that showed the opposite pattern, namely, their activity was significantly higher when the intervals were categorized as long (Supplementary Fig. 9a–f). Considering both monkeys, 134, 146, and 104 category-selective neurons showed higher activity for short intervals during T1, T2, and T3, respectively (Fig. 7c, and Supplementary Fig. 9). Of these, 89 neurons were category selective for two or more blocks of stimuli. In turn, 113, 92, and 71 category-selective neurons showed higher activity for long intervals during T1, T2, and T3 (Supplementary Fig. 9) and 64 neurons were category selective for two or more blocks of stimuli.

A further important feature is the moment at which these cells started to show category-selective activity, whereas, the neurons that preferred the short category increased their activity after the presentation of the second stimulus (Fig. 7a, c and Supplementary Fig. 9a-f), the long-preferring population tended to do so even before the interval offset. This effect, which was stronger in monkey 1, makes sense if, as we have argued, monkeys were comparing a representation of the block’s boundary to the interval presented on each trial: long intervals could have been correctly categorized as soon as the elapsed time exceeded the limit between categories, whereas a second stimulus arriving before the peak of the boundary neurons would signal a short category. Indeed, this temporal difference in the emergence of category-selective activity was also observed in our model (Fig. 6b) These results indicate that a large population of pre-SMA cells parametrically encoded the trial by trial categorical decision of the monkeys throughout the end of the stimulus period and the delay of the categorization task. Crucially, in these periods of the task the monkeys could not predict the location of the targets used to communicate their categorical decision. Therefore, the category-selective neurons were tightly related to the categorical choices of the monkeys without the contamination of the intention to perform a reaching movement toward a particular location.

### Neural activity related to the trial's outcome

**Fig. 8:**
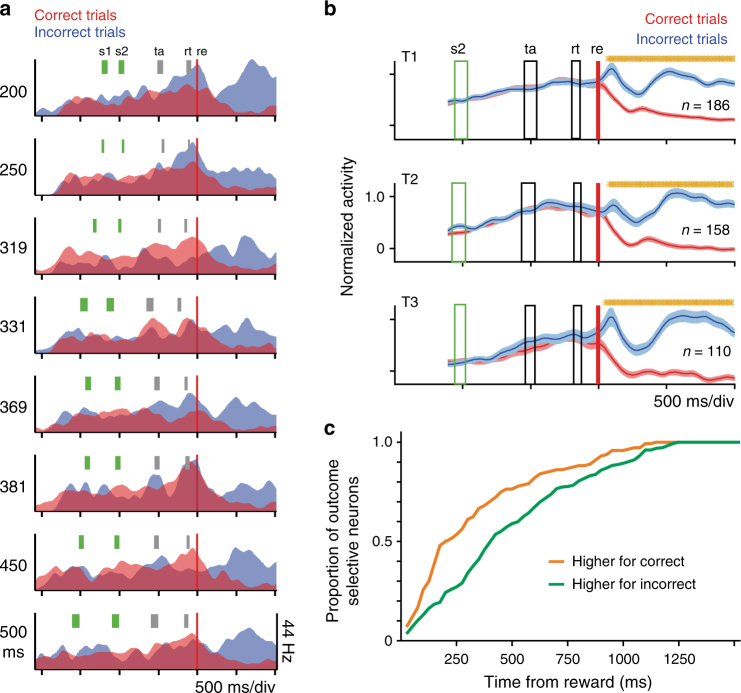
Decision outcome neurons. **a** Example of a pre-SMA neuron with higher activity for incorrect and unrewarded trials during the intertrial interval. The activity was separated into correct (red) and incorrect (blue) trials. s1, s2, ta, and rt indicate the standard deviation for first stimulus, second stimulus, targets and reaction time, respectively. **b** Population activity (mean ± SEM) of the neurons of monkey 2 whose firing rate is significantly larger for incorrect trials (blue function). Orange asterisks indicate: (1) *t*-test significant differences in non-overlapping sliding windows (over the population SDF, correct vs. incorrect, 95% bootstrap confidence interval, 100 iterations). The single cell (**a**) and the population activity (**b**) are aligned to the expected time of reward (re, red vertical line). **c** Cumulative distributions of the response onset latencies of the neurons selective for correct (orange) and incorrect (green) categorical decision outcomes during de intertrial interval. Note that the response onset latencies are shifted to the right for incorrect outcomes and are significantly larger for correct trials (Kolmogorov–Smirnov test, *p* < 0.0001)

Finally, we found neurons whose activity during the inter-trial interval (ITI) of the categorization task was modulated by the presence or absence of reward after the previous choice. These neurons were called outcome-selective cells and showed the following response properties: the outcome factor was significant (*p* < 0.05) in the permutation test of the multiple linear regression model described above, the outcome probability index, which represents the proportion of trials for which the outcome can be decoded from the neuron's activity (see ONLINE METHODS and Supplementary Fig. 10a–d), was above 0.6, and the *χ*^2^-test on the contingency table between the actual and decoded outcomes across all trials was significant (*p* < 0.05). Figure 8a illustrates the activity of an outcome-selective neuron whose activity was larger during incorrect trials for all the test intervals of block T1. The corresponding outcome probability index was 0.96 and the *χ*^2^-test was *x*^2^ = 66.79 (*p* < 0.001, 96 trials), supporting the notion that this neuron’s activity was tightly modulated by the trial outcome. Neurons with the opposite pattern of activity, showing higher activity during the ITI for correct trials, were also recorded (see the mean population response in Supplementary Fig. 11). From both monkeys, 293, 250, and 191 neurons showed higher ITI activity for incorrect trials in T1, T2, and T3, respectively. Of these, 232 neurons remained outcome-selective for two or more blocks of stimuli. In turn, 83, 100, and 56 neurons showed higher ITI activity for correct trials in T1, T2, and T3, respectively (Fig. 8b and Supplementary Fig. 11a-f) and 52 neurons maintained outcome selectivity for two or more blocks of stimuli. The number of incorrect-related neurons was statistically larger than the number of correct-related cells (*X*^2^_(1)_ =135.01, *p* < 0.001). A Kruskal–Wallis test was performed to determine whether the ‘outcome’ neurons showed additional selectivity for short or long intervals (Methods section). For both monkeys, 0% of the correct-selective outcome neurons and only the 0.74 % of the incorrect-selective outcome neurons showed statistical differences in their response for short and long intervals. Hence, there is no interaction between the neural encoding of the outcome and the categorical decision.

An important property of the outcome cells is that the correct-selective cells were recruited earlier than incorrect-selective neurons, which emphasizes a differential role of these two populations (Fig. 8c). Finally, it is important to mention that the outcome related activity was not associated with the oculomotor behavior of the monkeys during the inter-trial period. We employed a multiple linear regression model to account for possible eye position effects (see Experimental procedures, equation 2), and found that only a small percentage of outcome cells (4% monkey 1 and 5.47% monkey 2) lost their selectivity when considering the eye position behavior. In sum, these results suggest that some pre-SMA neurons maintained a representation of the consequences of the previous choice during the ITI, with a differential processing for correct and incorrect trials.

### Sequential organization of neural responses

**Fig. 9:**
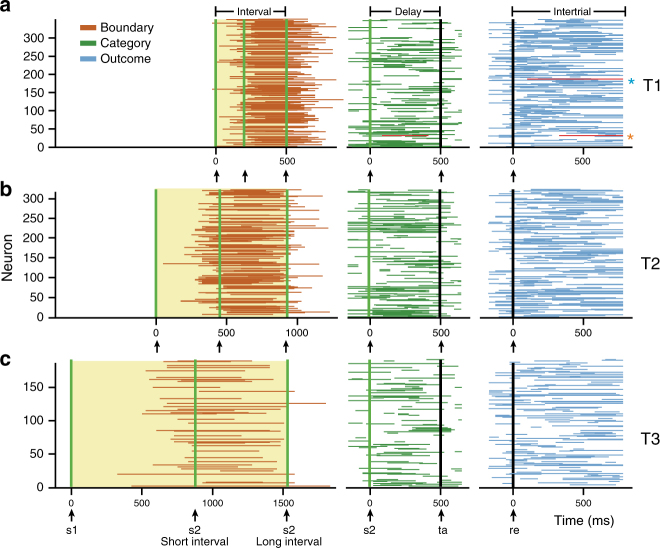
Neural activity encoding boundary, category, and trial outcome emerged sequentially throughout the task. Times at which pre-SMA neurons of monkey 2 carried significant boundary, category or outcome information for T1 (**a**), T2 (**b**), and T3. **c** Each row corresponds to a single neuron. Orange asterisk in **a** left indicates the neuron in Fig. 7a. Blue asterisk indicates neuron in Fig. 8a. Arrows at the bottom of each graph indicate task events. Boundary-related activity (left panel, brown) is aligned to the interval onset (s1); the offset of the shortest (s2 short) and longest (s2 long) intervals are indicated. Category-related activity (center panel, green) is aligned to the interval offset (s2). Outcome-related activity (right panel, blue) is aligned to reward delivery (re)

Throughout the trial, the neural representation of the categorical boundary, the category selected by the monkeys, and the trial outcome emerged sequentially in the population of pre-SMA neurons (Fig. 9a–c and Supplementary Fig. 12a-d). The boundary was encoded near the end of the interval presentation (Figs. 2a, b, 4a, b, 5, 9a–c). In turn, the category-related activity emerged at the end of the test intervals and during the delay (Figs. 7a, c, 9a–c, Supplementary Figs. 9a-f and 12a-d). Finally, outcome-related activity was observed during the movement times and the inter-trial period (Figs. 8a, b, 9a–c, Supplementary Figs 11a–f and 12a–d). While the subjective boundary was encoded through the moment of occurrence of the peak of activity, the category and outcome were represented by a firing rate code modulated by the corresponding task parameter. Notably, some individual neurons encoded more than one parameter (Supplementary Fig. 13). For example, the neuron in Fig. 7a showed increased activity for short categorical responses during the delay epoch and also showed a larger activation for incorrect trials during the intertrial period (the blue SDF in the 319 and 331 ms panels and the red SDF in the 369, 381, and 450 ms panels of Fig. 7a). Similarly, we found that some boundary neurons sequentially encoded more than one parameter. Supplementary Fig. 14, for an example, shows a boundary neuron with an additional selectivity for the categorical choice during the delay.

## Discussion

To clarify the neural mechanisms underlying flexible time categorization, we recorded the activity of pre-SMA neurons of Rhesus monkeys performing a relative interval categorization task. Given that there were three possible blocks of intervals, the task implied a redefinition of category memberships and boundaries every time a new block was presented. Notably, to avoid confounding neural activity related to motor planning/execution with that related to category membership, we carefully designed a way for the monkeys to communicate their decision without any particular movement direction being associated at all with a particular decision. Our psychophysics analysis indicated that monkeys indeed attended the duration of the test intervals to solve the task and successfully changed their categorical boundary within recording sessions to correctly categorize the test intervals of the different blocks of stimuli. Accordingly, the neurophysiological data showed that pre-SMA cells encoded the boundary between categories by reaching a constant peak of activity close to the limit between the short and long intervals. This implied that the location of this activity peak differed according to the location of the categorical boundary of the current block, and showed an activation profile that could explain the trial-by-trial categorical performance of the animals. Indeed, our analyses suggest that monkeys categorized the test intervals by comparing the neural representation of the categorical boundary with the time of occurrence of the stimuli indicating the interval offset. We demonstrated that a neural-network model following this strategy solved the categorization task and replicated the monkeys’ psychometric performance. Furthermore, the results showed that single cells in pre-SMA parametrically represented the category selected by the animals and the outcome of the monkeys’ decisions. All this information was encoded throughout the trial in a sequence that matched the cognitive requirements of the task. We discuss our results in a broader context below.

Boundary neurons encoded the boundary between short and long categories during the presentation of the test interval by reaching their peak activity at the subjective boundary between short and long intervals for a block of trials. Thus, within a trial block there was a systematic variation between the moment at which the activity peak was reached and the presentation of the second stimulus. For short test intervals, this activity peak tended to occur after the second stimulus; whereas, for long intervals it usually occurred before it, closely following the location of the monkey’s subjective limit between categories. Remarkably, at the population level, the internal boundary representation changed when monkeys categorized the intervals of different blocks of stimuli. Most of the cells encoded the boundary for only one block, although a subgroup of cells shifted their boundary representation in more than one block. Thus, the neural representation of boundary ‘moved’ along the ‘line of time’, adapting to the behavioral context. We hypothesized that an optimal reader can use the population activity of boundary cells and compare it to the occurrence of the second stimulus in order to categorize an interval in a specific block of stimuli. Whereas the information about the occurrence of the second stimulus can be a sensory one, as modeled here, this could also be a timing signal in the form of ramping neurons^30,35^ or cells tuned^32^ for intervals in the hundreds of milliseconds range, as has already been described in the pre-SMA. In fact, we observed some ramping neurons in this task as well (not reported). It is then likely that the interval duration coded by these cells, in conjunction with the population activity of the ‘boundary neurons’, allowed for the trial by trial decoding of the category selected by the monkeys. Our neural-network model demonstrated the feasibility of this mechanism.

The present findings agree with one of the most discussed theories for explaining perceptual categorization, the decision-bound theory (DBT)^1^, one version of which proposes that the stimulus is compared with a decision criterion that represents the subjective category boundary^1,46,47^. Previous studies using an interval categorization (bisection) task have suggested that such decision boundary corresponds to a value near the mean interval of the durations tested^20–22^. Since in our experimental design the value of the category boundary and the mean duration are correlated across the blocks of stimuli, it is difficult to entirely dissociate whether the boundary cells encoded the mean duration of a block or the subjective boundary. Nevertheless, two results support the hypothesis that the boundary cells encoded the subjective category boundary. First, from the activity of these cells we were able to decode with high fidelity the categorical decision made by the monkeys on a trial by trial basis. The key piece of information is the temporal relation between the peak of neural activity of the boundary cells and the timing of the second stimulus, and this occurred well in advance of the monkey’s motor response (for some trials 1500–2000 ms before the monkeys’ response). Additional support to this idea comes from the observation that the variation in the psychometric point of subjective equality from experiment to experiment was accompanied by a shift in the peak of the population of boundary cells, closely following the monkeys’ biases. In contrast, the stimulus mean duration remained constant. To our knowledge, this is the first time that a single-neuron correlate of a category boundary is described, which, moreover, is flexibly and quickly adapted to changing category definitions within a single recording session^6,7^. These results provide neurophysiological support to many psychophysics and EEG studies in humans in favor of the DBT for time categorization^21,22,47^.

Based on our neurophysiologic observations, we built a model that implements the categorization process as a mechanism that selects the short category when the neural response to the second stimulus occurs first, or the long category when the peak activity of the boundary cells occurs first. In latter case, the long category signal should emerge before the presentation of the second stimulus. Several anatomical and neurophysiological studies point to the neostriatum, specifically the putamen, as the best candidate for implementing this comparison. First, the putamen interacts with pre-SMA through the motor cortico-basal ganglia-thalamo-cortical circuit (CBGTc), a circuit involved in the control of voluntary skeletomotor movements and in the perceptual and motor aspects of timing^31,34,48–52^. Second, the putamen has privileged access to visual information since it receives inputs from almost all visual cortical areas^53^ and from regions of the thalamus in which a high proportion of neurons signal the onset of behaviorally relevant visual stimuli^54^. Third, several studies have shown transient striatal neural responses to behaviorally relevant visual stimuli with latencies as short as 40 ms^55,56^. Therefore, the putamen is the area that could be performing the comparison between the short latency neural response to the second stimulus and the activity of the boundary cells, and could be sending the categorical signal back to the pre-SMA through the CBGTc. Future simultaneous recordings in the striatum and pre-SMA are needed to test our hypothesis. A second possibility is that this comparison is done in the medial premotor cortex itself, using a more complex timing signal instead of a sensory one. In this case, the activity of neurons that ramp as a function of the interval duration^30,35^ could compete against that of the boundary cell population, giving rise to a short decision if the ramping neurons reach their peak first or to a long decision otherwise.

We also found single-pre-SMA neurons that showed category-selective activity that allowed the trial by trial decoding of the monkey’s responses. We observed neurons tuned to both short and long categories. This category-selective activity was mainly observed during the delay epoch. Nevertheless, at the population level, strong long-category selectivity emerged before the interval offset. In contrast, short-category selectivity was more robust after the interval offset. These findings suggest that monkeys were able to assign the category membership before the offset of the long-test intervals, a hypothesis that has been also proposed for humans^22^. Moreover, the mean latencies of category-selective activity for the long intervals were similar to the mean times of peak activity of the boundary neurons, supporting the proposition that the neural boundary was employed for deciding the category membership of the intervals. Additionally, it is important to emphasize that the observed category-related activity cannot be attributed to motor planning or execution, given that in our task, the communication of the categorical decision was not tied to a particular response direction. Consequently, monkeys could not plan the response movement until the end of the delay, once targets had been presented, whereas the category-related activity emerged well before target presentation.

Finally, we found that during the inter-trial period, the activity of some neurons discriminated correct from incorrect trials, an observation that has been reported for other cortical areas as well^57,58^. We hypothesize that the signal conveyed by these cells could have an effect on the current representation of the neural boundary, which should be reflected in the monkey’s trial by trial performance. Neurons with larger activity for correct trials were recruited earlier than incorrect-selective neurons, probably because their activity was associated to reward delivery. However, the population of neurons responding to incorrect trials was significantly larger, suggesting that more weight was being given to this signal for the trial by trial adjustment of the subjective between-categories boundary. Neural-network models of categorization have conferred great importance to reward information^23^ and future studies will shed light on our hypotheses. Our observation coincides with intra-cerebral recording, fMRI, and ERP studies showing that the SMA/pre-SMA is part of a frontal-medial system for the evaluation of the outcome of actions^59–63^.

In sum, we demonstrated that single-pre-SMA neurons sequentially encoded all the relevant information needed for solving the interval categorization task. During interval presentation, we found signals representing the subjective boundary needed for assigning the different intervals to the corresponding categories. Importantly, we provided a putative mechanism for how this boundary representation was employed by the brain to categorize the different intervals. In addition, we observed that single-pre-SMA neurons represented, during the delay and inter-trial periods, respectively, the category selected by the monkeys and the outcome of the perceptual decision. Finally, we propose that the consequence of the monkey’s decision, encoded by outcome-related neurons, could serve to adjust the neural boundary representation in order to improve task performance. We will analyze this possibility in future reports.

## Methods

### Animals

Two male Rhesus monkeys (*Macaca mulatta*): monkey 1 (5.5 kg BW) and monkey 2 (7.2 kg BW) were tested. All the experimental procedures were approved by the National University of Mexico Institutional Animal Care and Use Committee and conformed to the principles outlined in the Guide for Care and Use of Laboratory Animals (NIH, publication number 85–23, revised 1985).

### Materials

During task performance, the monkeys were seated in a primate chair with their head and left arm restrained. The gaze position was measured with an infrared tracking system (ISCAN, Inc., Woburn, MA, USA). Visual stimuli were presented in a computer monitor (HP7540, 160 Hz refresh rate) 56 cm away from the monkey’s eyes. The task required that monkeys manipulated a joystick (H000E-NO-C, CTI electronics, Stratford, CT, USA) to control the position of the cursor on the screen.

### Task

The details of the task have been presented previously^2,26^. Briefly, monkeys were trained to categorize the interval between two visual stimuli as either ‘short’ or ‘long’ according to previously learned prototypes. Figure 1a shows the temporal sequence of a trial. A circle containing a fixation point was shown in the center of the screen. The animal started the trial by staring and keeping his gaze within a circular window with a diameter of 4° of visual angle which was centered at the fixation point and by placing and maintaining the cursor inside the central circle. After a variable waiting period (500 + Δ 1000 ms) two parallel bars (8° × 0.7° of visual angle) separated by constant distance (6° of visual angle) appeared briefly (50 ms), disappeared during a particular test interval, and reappeared in the same position. The first and second stimulus presentation indicated, respectively, the beginning and the end of the test interval. While the duration of the test interval changed from trial to trial, the position of the bars was always the same. After a fixed delay (1000 ms for monkey 1 and 500 ms for monkey 2) two response targets (orange and blue circles), were presented (Fig. 1a, b). Monkey 2 had a lower performance with a delay of 1000 ms, hence, we set it at 500 ms in this animal. Importantly, across trials, both response targets could occupy one of eight possible locations on the periphery of the screen, which precluded the contamination of the categorization process by the intention to move to a particular place in space (Fig. 1b). The monkeys were trained to move the cursor from the central circle to the orange target if the test interval was short or to the blue target if it was long. The monkey received a juice reward immediately after correct responses. The amount of reward was adjusted to be proportional to the block of durations being categorized (see ref. ^2^). The inter-trial interval (ITI) was 1500 ms. Eye fixation was enforced from the beginning of the trial until target presentation, when monkeys were allowed to break fixation.

### Stimuli and task procedures

Three blocks of stimuli (T1, T2, and T3), each containing eight different intervals and different between-categories boundaries, were employed. The first four intervals of every block were considered “short” (Fig. 1c) and the remaining “long”. Furthermore, some durations were present in two blocks but belonged to a different category in each case, emphasizing the context-dependent nature of categorization. Consequently, within recording sessions, the monkeys were forced to flexibly change their subjective limit between categories to successfully categorize the intervals of different blocks. All the intervals of each block were randomly (monkey 1) or pseudo-randomly (monkey 2) presented. For every block of stimuli, the monkey had a training and a testing phase. The first 24 trials of a block of trials constituted the training phase in which only the shortest and the longest intervals of each block were presented in an alternate fashion (12 repetitions per interval). Importantly, in this phase the color of the stimulus bars was orange when the short interval was presented and blue for the long interval. Consequently, it matched the color of the correct response target, and defined the short and long prototypes to be memorized for this block. The following 96 trials constituted the test phase in which every one of the eight stimuli of the current block was presented 12 times. Crucially, the color of the stimulus bars during this phase was green regardless of the stimulus category, so the animals required to remember the prototypes and/or an implicit limit or boundary interval to solve the task. In every recording session, the three test blocks (T1, T2, and T3) were randomly presented to the monkey.

### Surgery

Recording chambers (8 mm inner diameter) were implanted over the left pre-SMA and DLPF cortex of monkey 1 and 2 during aseptic surgery under Sevoflurane (1–2%) gas anesthesia. In monkey 1, after recording the neural activity in the left hemisphere, the chambers were surgically moved to the right pre-SMA and DLPF cortex. Chamber positions were determined on the basis of structural MRI (Supplementary Fig. 2). Titanium posts for head restraining were also implanted. Broad spectrum antibiotics (Enrofloxacin, 5 mg/kg/day, i.m.) and analgesics (Ketorolac 0.75 mg/kg/6 h or Tramadol 50–100 mg/4–6 h, i.m.) were administered for 3 days after surgery.

### Neuronal recordings and spike sorting

The extracellular activity of neurons in the pre-SMA was recorded with quartz-insulated tungsten microelectrodes (1–3 MΩ) mounted in multielectrode manipulators (Eckhorn System, Thomas Recording, GMbH, Giessen, Germany). All neurons were recorded regardless of their activity during the task and the recording site changed from session to session. Spike waveform data were sorted off line (Plexon Offline Sorter, v3.0. Plexon Inc. Dallas, TX, USA, monkey 1) or online employing window discriminators (Blackrock Microsystems LLC, Salt Lake City, UT, USA, monkey 2).

### Behavior

For every monkey and block of trials, we calculated a psychometric curve as the logistic fit to the probability of categorizing each interval of the corresponding block of stimuli as long (‘*p* long’, all fits with *p* < 0.05). From every curve, we calculated the difference limen (DL) as half the subtraction of the interval at ‘*p* long’ = 0.75 and at ‘*p* long’ = 0.25. Similarly, the point of subjective equality (PSE) was the interval at ‘*p* long’ = 0.5 ^64^. The CE was computed as the difference between the PSE and the implicit (theoretical) limit between categories for every block of stimuli. Additionally, we computed the reaction time defined as the interval between the time of presentation of the response circles and the moment in which the monkey moved the cursor out of the central circle.

### General neural activity

Subroutines written in Matlab (Matworks v. 7.6.0.324) and the SPSS statistical package (version 20, SPSS Inc., Chicago, IL, 2011) were used for the statistical analyses. The level of statistical significance to reject the null hypothesis was *α* = 0.05.

### Cell stability

We used previously validated criteria to assess the single-unit stability during the performance of the different blocks of the time categorization task by measuring the similarity of the average spike waveforms and the interspike interval histograms (ISIHs)^31,42^. The stability threshold used was 10.5, which was reported as an appropriate value in chronic single-cell recordings^42^. From the total of cells (259 of Monkey 1 and 620 of Monkey 2), 816 cells showed a similarity score*I* below the 10.5 threshold in at least two consecutive blocks (196 of monkey 1 and 620 monkey 2), and were considered stable for these blocks.

### Up–down activity around the test interval

We used an iterative algorithm to detect the up–down profile of instantaneous activity for each interval of the three blocks. This algorithm has the following steps. Step 1: spike times were convoluted with a Gaussian kernel (*σ* = 30 ms) to obtain the SDF for each trial, including the 500 ms control period, the test interval, and 500 ms of the delay period (Fig. 3a). Step 2: SDF was aligned to the first stimulus presentation. Step 3: the time of the activity peak was identified along the interval and delay periods (Orange circle Fig. 3b). Step 4: the minimum activity time was found (Green circles, Fig. 3b), and a regression was performed between the minimum and peak times. The activity minimum could be located before or after the peak, defining ramps with positive or negative slopes, respectively (Fig. 3c). Step 5: regressions were carried out decreasing, in steps of 20 ms, the interval from the minimum activity to the peak time (Blue lines Fig. 3b). Step 6: we considered that the algorithm reached convergence when the regression R^2^ decreased by less than 5% on subsequent iterations. Step 7: The best regression model in terms of adjusted *R*^2^ was found (Black lines Fig. 3c). Step 8: a ramp was defined when its peak was above 8 Hz, the duration was longer than 100 ms, and the linear regression had a *p* < 0.01. Finally, a cell was considered a boundary neuron if it fulfilled three conditions: (1) an up–down ramp was detected in at least 5 of the 8 test intervals of a block using the SDF across trials; (2) the magnitude peak of the SDF across trials was above 8 Hz; and (3) the peak activity of the cells showed no significant effects on *τ* as a function of test interval (ANOVA, *p* > 0.05), computed from the trial by trial up–down activity. We performed the previous analysis using different analysis windows: (1) an analysis window including the categorized interval plus 500 ms after the second stimulus, (2) a constant window of 700 ms before and after the second stimulus across durations and blocks on the original boundary cells, and (3) a window of fixed size across test intervals (the longest test interval of each block plus 500 ms before and after) and aligned to the first stimulus.

It is important to notice that using a window of constant length across test intervals and blocks that includes the largest test interval of the block T3 (i.e., 1520 ms) yields uninterpretable results for block T1. The shortest interval of T1 is 200 ms; consequently, a detection window of 1520 ms covers the test interval, the delay (which in monkey 1 is 1000 ms and in monkey 2 is 500 ms) and large part of the reaction time and movement time periods of the task, especially in monkey 2 (see Fig. 1 and Supplementary Figure 1). Consequently, the utilization of windows of constant size across blocks will lead to the detection of responses that could be associated with the categorical boundary, the categorical decision, the targets presentation, and the preparation and execution of the reaching movement in T1. Crucially, the results from the new analysis of block T3 support the notion of a shift in the activity peak of boundary cells across blocks. The analysis in this block included a constant analysis window of 500 ms before (control period) to 2020 ms after the first stimulus. In this case, the analysis had the sensitivity to detect activity peaks that are quite smaller than the values around the implicit interval (1195 ms). Yet, the algorithm found peaks above 768.8 ms, with no overlap with the peak time obtained in the block T1 (T1 mean and SD: 424.56 and 64.91 ms; T3 mean and SD: 1088.01 and 136.04 ms; Supplementary Fig. 5b). Indeed, with this analysis we found 24 neurons that were considered boundary cells in blocks T1 and T3 which showed significant shifts of activity peak between blocks (Wilcoxon signed-rank test; *p* < 0.05).

On the other hand, in order to test whether a cell encoding the boundary in one block was likely to encode the boundary in the other blocks in which it was stably recorded, we performed a *χ*^2^-test on a contingency table. We asked the question “if one neuron is a boundary cell in a block T(small), is the same neuron likely to be a boundary cell in a block T(large)?”. Neurons stably recorded in 3 blocks contributed 3 comparisons: T1 vs T2, T1 vs T3 and T2 vs T3.

### Decoding category membership from boundary neuron's activity

We constructed neurometric curves employing the activity of boundary neurons to categorize intervals as short or long in a trial by trial basis. We first quantified the elapsed time (*τ*) between the peak of activity and the occurrence of the second stimulus for each of the trials with the eight test intervals. Next, we found the value (decoding criterion) that minimized the error to classify the stimuli as short or long short based on *τ*. Finally, a neurometric curve was constructed by computing the probability that the neuron chose a test interval as “short” or “long” based on the number of trials that were above or below this decoding criterion (Fig. 4c–e). A contingency table and a *χ*^2^-test was performed between decoded and the observed monkey’s choices across all trials. The level of significance to reject the null hypothesis was *α* = 0.05. We also computed an analog of the choice probability index^43,44^, which indicates the proportion of behavioral responses that can be predicted from *τ* values. This index acquires values between 0 and 1; where 1 indicates a perfect separation between the *τ* distributions for short and long responses. This choice probability index was “signed”, namely, positive *τ*s were associated with a short choice and negative *τ*s were associated with a long choice, producing a choice probability >0.5 (Fig. 4d). A ROC curve was calculated from the neural and behavioral data of the four intermediate intervals in a block, those with a balanced relation between short and long categorical decisions. All neurons with a Choice Probability index larger than 0.6 were considered selective. Alternatively, for each cell and block of stimuli, we also calculated a mean from the indexes calculated separately for each of the four intervals. A paired *t*-test did not find significant differences between the indexes calculated by the two methods (indexes from grouped intervals: mean = 0.698 SD = 0.077; indexes from individual intervals: mean = 0.686 SD = 0.081; *t*(129) = 1.59 *p* = 0.11).

### Neural selectivity to interval, categorical choice, and reward outcome

The activity of the stable cells was subjected to two analyses. The first one was an ANCOVA that used the discharge rate during the delay, the reaction-movement time, or the intertrial period as the dependent variable, the discharge rate during the key holding control epoch as the covariate, and the test interval, categorical choice (short/long), and reward outcome (correct/incorrect) as the factors.A total of 685 cells (164 monkey 1 and 521 monkey 2) showed significant effects in at least one factor and for at least one task epoch. These cells were analyzed further in the following multiple linear regression model using sliding windows of 250 ms with steps of 25 ms:

1 $$r = \beta _0 + \beta _1{\mathrm{M}} + \beta _2{\mathrm{C}} + \beta _3{\mathrm{O}}.$$

where *r* is the discharge rate of the cell, M is the duration of the test interval, C is the categorical choice (short/long), and O is the reward outcome (correct/incorrect). We used a permutation test (100 iterations) to determine the significance level for each coefficient in the model. For each neuron, neural responses were permuted 100 times across the 12 trials and 8 test intervals for each sliding window. The multiple regression was computed for each permutation to obtain a ‘null’ distribution of *p*-values. The multiple regression was considered significant if the non-permuted *p*-value was lower than the *p*-value from the ‘null’ distribution at 95% of confidence. The sliding windows were run through the delay with cell responses aligned to the second stimulus, through the reaction and movement time aligned to the target presentation, and during the intertrial period aligned to the reward time. A cell was considered to have a significant effect from one of the coefficients if the permutation test of two consecutive sliding windows was *p* < 0.05. It is important to mention that a detailed collinearity analysis (SPSS collinearity tests) was performed between M, C, and O, with no evidence of collinear interactions between the three factors.

We calculated two additional measures of the association between neural activity and the monkey's choices for the same sliding windows employed in the multiple linear regression model: (1) the choice-probability index indicates the proportion of behavioral responses that can be predicted from the neuron's activity^43,65,66^. This measure quantifies the overlap between two neural response distributions, in this case between short and long responses. For each neuron, we constructed two firing rate distributions, one associated to short and another associated to long responses. We restricted the analysis for the intermediate intervals, for which the animals had a probability of error between 0.3 and 0.7 (i.e., the third to sixth time intervals of each block, see Fig. 1c, d). We employed these distributions to calculate an ROC curve. A resulting value of 0.5 indicates a full overlap, whereas a value of 1 indicates a complete separation between the short and long response distributions. Therefore, the choice-probability index determines the selectivity of a neuronal response to short or long categorical decisions. All neurons with a choice probability larger than 0.6 were considered selective. (2) In addition, a contingency table and a *χ*^2^-test was employed to measure the interrelation between the monkey’s actual response and the response decoded from the discharge rate of a cell across all trials was calculated as follows. For every neuron and block of trials we found the FR value (criterion) that best delimited the FR distributions associated to short and long choices. Next, the criterion was employed to categorize each trial as ‘short’ or ‘long’ on the basis of the corresponding observed neural activity. Finally, a *χ*^2^-test was performed on the contingency table calculated between the decoded and the observed monkey’s choices across all trials a block. The level of significance to reject the null hypothesis was *α* = 0.05.

For neurons with a significant effect on the trial outcome, it was necessary to account for the neural signals related to eye position since monkeys could move their eyes freely after response circles presentation. For that purpose, we carried out a multiple linear regression analysis between the time-varying single cell activity and eye position. The model was de following:

2 $$f_{t - \varDelta } = \beta _0 + \beta _ix_t + \beta _2y_t,$$

where *f*_*t*_ is the SDF at time *t–Δ*, with *Δ* from 0 to 200 ms, *β*_0_ is a constant, *β*_1_ is the regression coefficient of the *x* coordinate of the eye position at time *t*, *β*_2_ is the regression coefficient of the *y-*coordinate of the eye position^67^. For outcome cells with significant regressions (*p* > 0.05), we evaluate the relation between the residual (with respect to eye position) and the magnitude of stimulus, the selected category and the trial's outcome in the multiple linear regression model described in equation (1). Only 14 from 124 outcome selective cells in monkey 1 and 34 from 392 outcome selective cells in monkey 2 showed significant effect of eye position. From these, 4 cells from monkey 1 and 23 from monkey 2 lost outcome selectivity after accounting for the effect of eye position.

On the neurons with a significant effect of the trial outcome according to the multiple linear regression, we calculated a measure analogous to the choice-probability index: the “error-probability index”. This index measured the distance between the FR distributions associated to correct and incorrect trials. In addition, we performed a *χ*^2^-test on the contingency table calculated between the actual response outcomes (correct/incorrect) and the outcome decoded from the neural activity across all trials of a block (Supplementary Fig. 9). To test whether outcome neurons show additional category selectivity, a Kruskal–Wallis test and a Dunn-Sidak post hoc test compared their mean firing rate for correct/short, correct/long, incorrect/short and incorrect/long trials. In this analysis, the mean firing rate of the bins with outcome related activity during the inter-trial interval was employed. The level of significance to reject the null hypothesis was *α* = 0.05.

### Neural-network model

Two independent networks composed of 800 excitatory and 200 inhibitory integrate-and-fire neurons were simulated. The cells were sparsely randomly interconnected with a probability of 0.05, and the synaptic weights between neurons were randomly distributed (Fig. 6a, center). Both networks received input pulses that drove: (1) an AMPA excitatory current that could trigger a paired-pulsed facilitation process, and (2) a GABAb slow inhibitory current. We included these currents since neural networks, including them have the ability to process time information in the range of hundreds of milliseconds^68^.

The sensory network received two pulses separated by one of the test intervals of the categorization task. The boundary network also received two pulses, but in this case the time between the pair of pulses was drawn from a Gaussian distribution mimicking the up–down profile of activation of the boundary cell population (Fig. 5).

PCA was used to reduce the high-dimensional activity of the recurrent network activity^69^. Thus, instead of using time-varying responses of sensory and boundary networks, we used the first principal component and the two principal components of the sensory and boundary network responses, respectively. PCA was computed on the binned activity (10 ms bins) for the excitatory neurons of both networks separately, using the responses across the eight test intervals. The resulting neural trajectories were analyzed using a linear classifier in order to determine whether a test interval was short or long based on the path of the trajectory for each simulation (total simulations 50; Fig. 6a right; Supplementary Fig. 8d). Thus, neural population trajectory of each simulation was categorized as long if the linear combination:

3 $$w_{\mathrm{s1}}r_{\mathrm{s1}}\left( t \right) + w_{\mathrm{b1}}r_{\mathrm{b1}}\left( t \right) + w_{\mathrm{b2}}r_{\mathrm{b2}}\left( t \right) + c > 0,$$

and short otherwise (Fig. 6a right). *r*_s1_, *r*_b1_ and *r*_b2_ are the trajectory values at time *t* of the first PC from the sensory and the two PC from boundary network, respectively. *w*_s1_, *w*_b1_, *w*_b1_ and *c* were the sensory, boundary, and constants weights^70^, respectively. Therefore, the linear classifier determined the plane in the PCA space that could divide short and long intervals according to equation (3). The discrimination plane (gray plane Fig. 6a right; Supplementary Fig. 8d) allowed to compute the probability of long interval classification based on the activity of the two networks in the PCA space (Supplementary Fig. 8d). Thus, networkmetric functions at time (*t*) were built using the probability of long interval categorizations by the linear classifier across simulations for each test interval (Fig. 6d). We optimized the time *t* (called threshold decision time; black arrow in the inset of Supplementary Fig. 8a), as well as the *w*_s1_, *w*_b1_, *w*_b1_ to minimize the mean squared error between networkmetric and psychometric functions across all test intervals. Since the threshold decision time was close to the categorical boundary, the classifier plane for short test intervals occurred after the stimulus presentation, namely, before the threshold decision time (Supplementary Fig. 8b). In contrast, for long test intervals the classifier plane occurred at the threshold decision time (Supplementary Fig. 8c).

### Neuron model

Neurons were modeled as Integrate-and-Fire units. Each cell was characterized by the membrane potential *V*. When *V* reached the threshold value of *V*_*t*_ = 20 mVan action potential was triggered, which was followed by a membrane potential of *V*_*r*_ = 0 mV during a refractory period of *t*_*r*_ = 1 ms. The membrane potential dynamics followed the equation:

4 $$\tau \frac{{{\rm d}V}}{{{\rm d}t}} = - V + I_{{\rm Fac}} - I_{{\rm GABAb}} + I_{{\rm rec}} + N,$$

where *τ* is the time constant of the neural membrane^71,72^, which was *τ* = 5 for excitatory neurons and *τ* = 10 for inhibitory neurons. *I*_Fac_, *I*_GABAb_ were the driving input currents that provided information about the test interval to each neuron. *I*_rec_ is the recurrent current in network and included a AMPA and GABAa components, corresponding to the excitatory and inhibitory recurrent inputs of the networks^73^. Finally, *N*(*t*) is the white noise of the system with a standard deviation *σ*_*N*_.

### Input currents

Input currents changed the membrane potential for each cell with temporal dynamics that obeyed the following equations:

5 $$\tau _{\mathrm{r,type}}\frac{{{\rm d}I_{{\rm type}}}}{{{\rm d}t}} = - I_{{\rm type}} + R_{{\rm type}}$$

6 $$\tau _{\mathrm{d,type}}\frac{{{\rm d}R_{{\rm type}}}}{{{\rm d}t}} = - R_{{\rm type}} + w_{{\rm type}}\delta \left( {t - t_{{\rm type}}} \right),$$

where *I*_type_ is the current and *R*_type_ is a recovery variable. *τ*_r,type_ and *τ*_d,type_ are the rise and decay time constants of each current type, and *w*_type_ is the synaptic efficacy of the input stimulus presented at time *t*_type_. The driving input currents were AMPA and GABAb; whereas, the recurrent currents were AMPA and GABAa. The time constants were: *τ*_r,AMPA_ = *τ*_r,GABAb_ = 0.2, *τ*_d,AMPA_ = *τ*_d,GABAb_ = 0.7, *τ*_r,GABAb_ = 21 and *τ*_*d*,GABAb_ = 500 ms. Each synaptic current was activated by action potentials, represented in the form of Dirac *δ* functions^74^.

Each synaptic current was activated by two pulses separated by the test interval for the sensory network and a Gaussian-distributed interval for each cell and trial of the boundary network.

### Short term plasticity

The model also included plastic facilitation processes, where the amplitude of the synaptic efficacy of AMPA currents changed upon the repeated presentation of stimuli^75^. This property can be modeled as a release probability *p*_rel_(*t*). Between pairs of stimuli, this probability follows the equation:

7 $$\tau _P\frac{{{\rm d}p_{rel}}}{{{\rm d}t}} = P_0 - p_{{\rm rel}},$$

where *τ*_*P*_ = 700 ms is the time constant and *P*_0_ = 0.17 is the stable release probability. In this model, *p*_rel_ changed its value immediately after of the appearance of an input action potential following the rule $p_{{\rm rel}} \to p_{{\rm rel}} + f_P\left( {1 - p_{{\rm rel}}} \right)$ (*f*_*P*_ = 0.62). Therefore, the synaptic weight in equation (−5) for current *I*_Fac_ was multiplied by factor $\frac{{p_{{\rm rel}}}}{{P_0}}$ (see ref. ^76^).

### Data availability

The data that support the findings of this study are available from the corresponding author upon reasonable request.

## Electronic supplementary material

Supplementary Information(PDF 1850 kb)
