# Supplementary material for: Neural basis for categorical boundaries in the primate pre-SMA during relative categorization of time intervals
Source: Nat Commun. 2018 Mar 15;9:1098. doi: 10.1038/s41467-018-03482-8 (PMC5854627; doi:10.1038/s41467-018-03482-8)
Supplement: Supplementary file 1 — Supplementary Information(PDF 1850 kb) [file 41467_2018_3482_MOESM1_ESM.pdf]

Neural basis for categorical boundaries in the primate pre-SMA during relative categorization of time intervals

Mendoza et al.

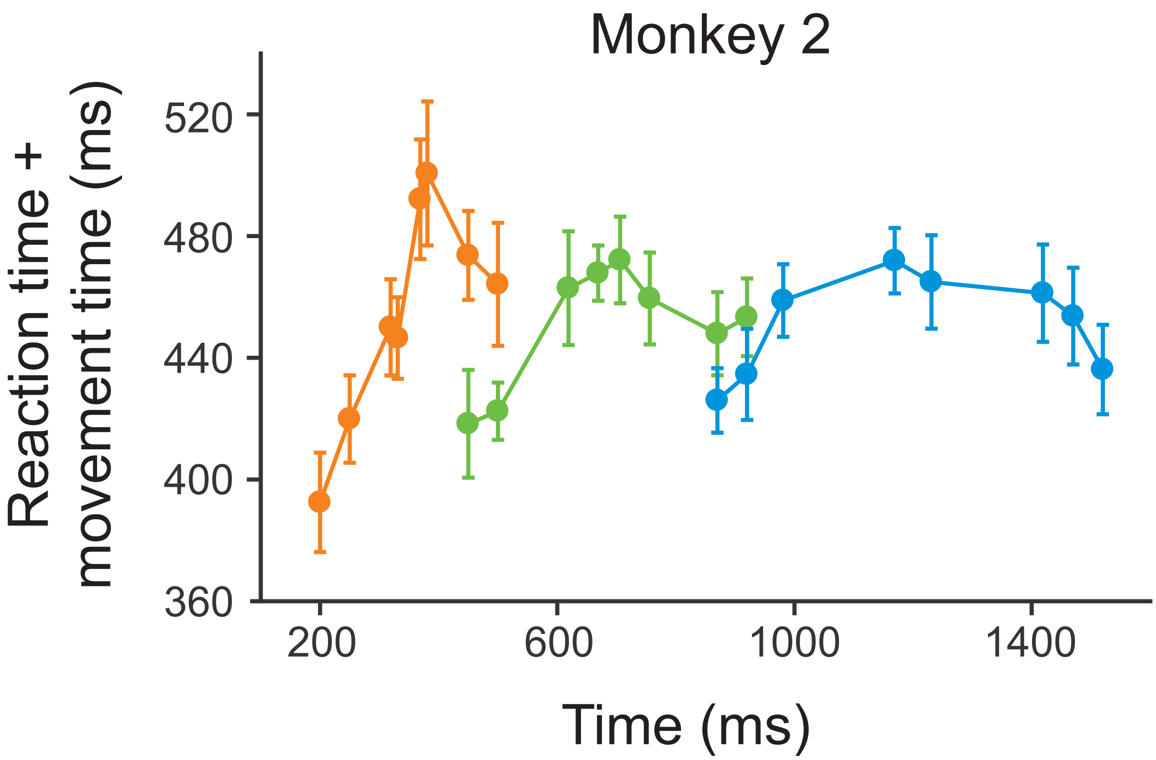

Supplementary Figure 1. Mean (± SEM) reaction-movement times of monkey 2 as a function of the test intervals for the three blocks of stimuli.

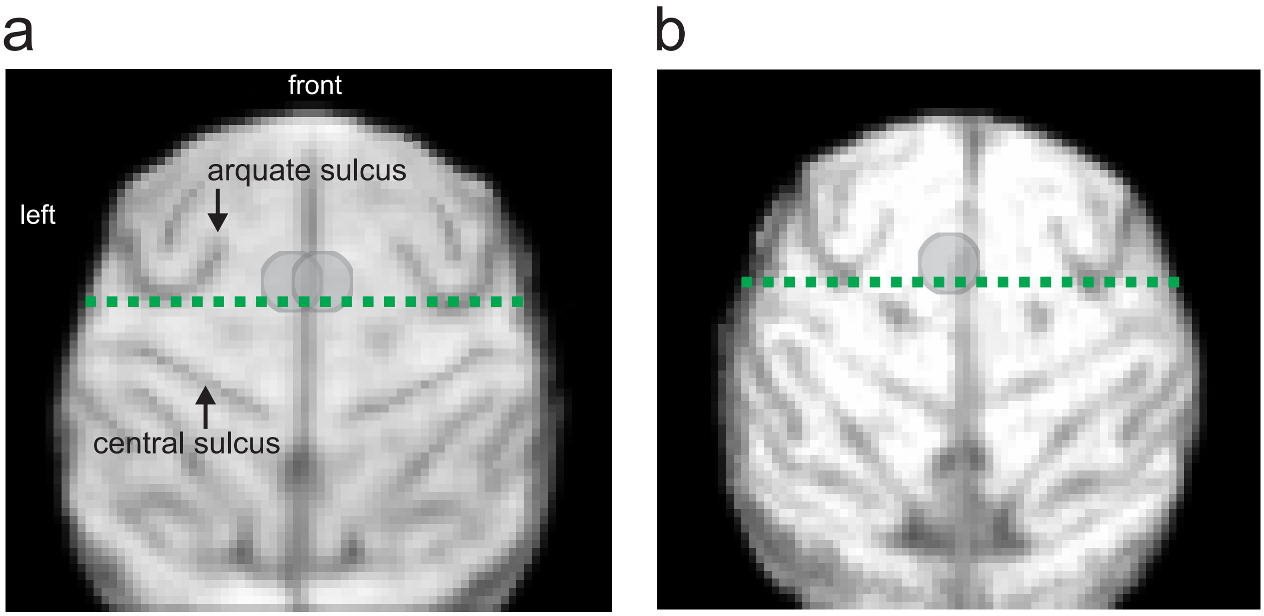

Supplementary Figure 2. Recording sites. Surface reconstructions of the brain sulci of monkey 1 (**a**) and monkey 2. (**b**) where the perimeter of the recording chambers on the left and right hemispheres is shown with a dark gray circle. In monkey 1, after recording left pre-SMA, the recording chamber was surgically moved to record the right pre-SMA. The horizontal line corresponds to the limit between pre-SMA and SMA and indicates the antero-posterior level of the genu of the arcuate sulcus.

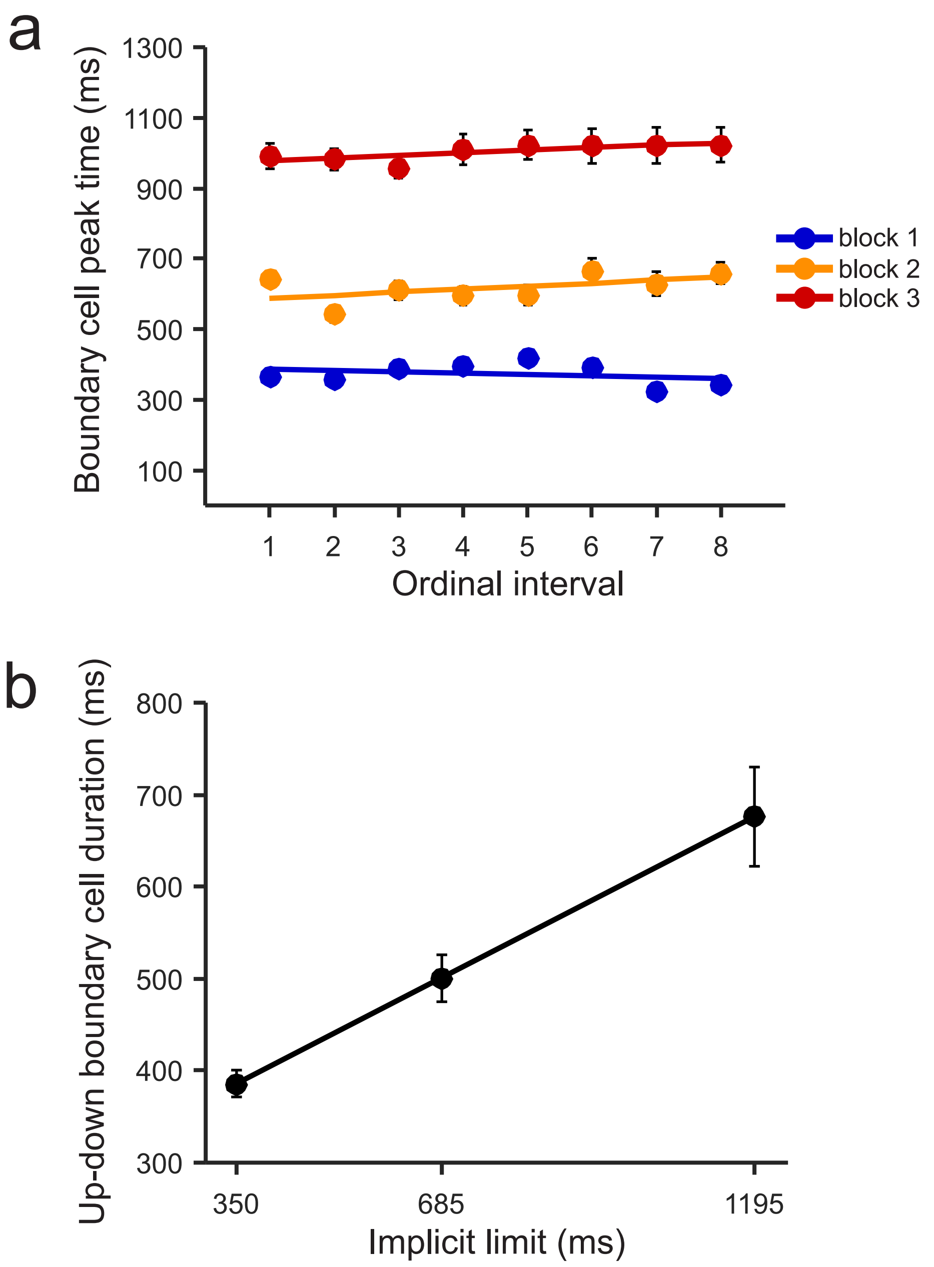

Supplementary Figure 3. The moment of occurrence of peak activity and the duration of the up-down activation of boundary neurons changes across different blocks of stimuli but not across the intervals within a block of stimuli. (**a**) Time of the occurrence of boundary neuron peak activity related to interval onset as a function of the ordinal value of the test intervals for T1 (blue), T2 (orange) and T3 (red). (**b**) Duration of the total up-down pattern of activity of boundary neurons as a function of the implicit limit of the tree blocks of stimuli (T1, T2, T3).

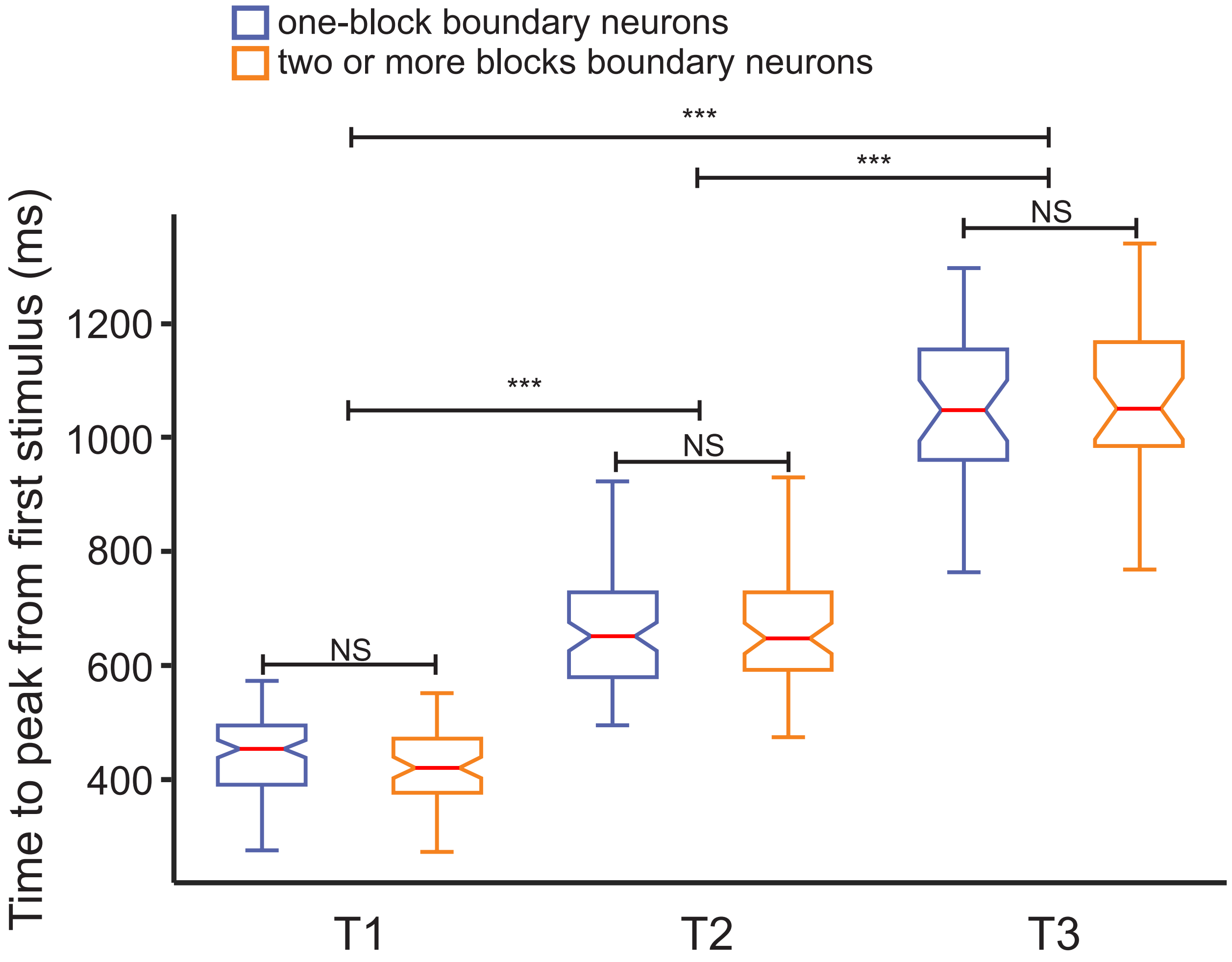

Supplementary Figure 4. Neurons encoding the boundary showed a shift in their time to peak activity according to the current block of intervals. Time to peak from first stimulus as a function of the three blocks of intervals for neurons considered 'boundary-related' during only one block (blue) or during two or more blocks (orange). It is evident that the population of boundary neurons adapted its peak activity according to the current block of intervals. Within every block of stimuli, a Kruskal-Wallis test and Dunn-Sidak post hoc test showed no significant differences between the time to peak of 'one-block boundary neurons' and 'two- or more blocks boundary neurons', whereas they showed significant differences between the time to peak across blocks ($\chi 2(5) = 312.62$, $p < 0.001$).

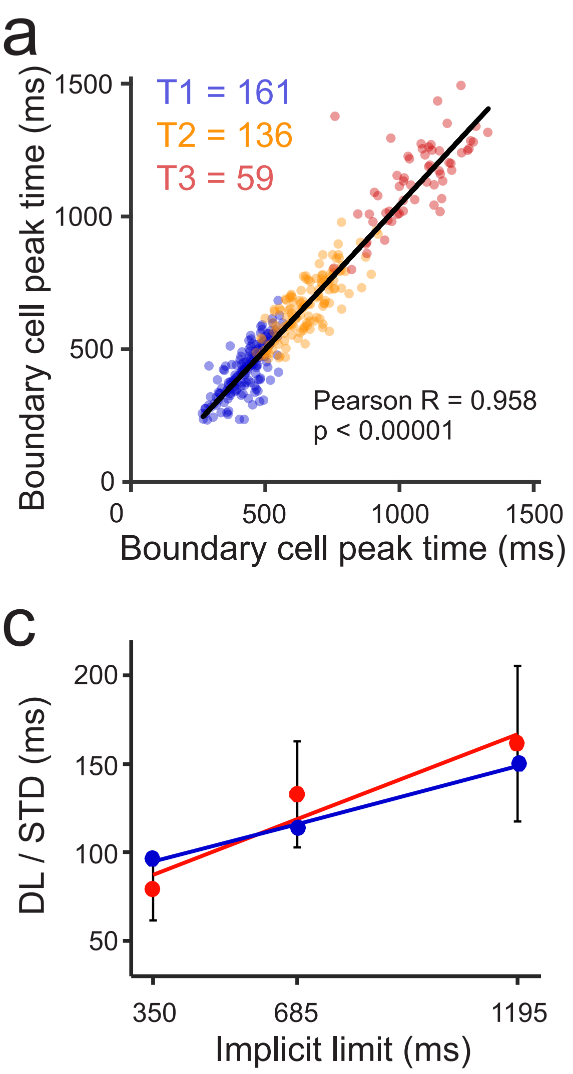

Supplementary Figure 5. (**a**) Correlation between the times of peak activity of boundary neurons obtained using a different window size across blocks and test intervals (starting 500 before the first stimulus and ending 500 ms after the second stimulus; abscissa) versus a fixed window of analysis of 700 ms before and 700 ms after the second stimulus (ordinate, see METHODS). The color of the dots corresponds to the block of the boundary neurons as follows: blue for T1, orange for T2, red for T3. (**b**) Correlation between the times of peak activity of boundary neurons obtained using a different window size across blocks and test intervals (as in a; abscissa) versus a window of a fixed size within the test intervals of a block (starting 500 before the first stimulus and ending 500 ms after the longest test interval of a block; abscissa). (**c**) DL (mean ± SEM) from both monkeys (red) and the standard deviation of the Gaussian functions of the peak time of the boundary neurons (blue) found with fixed analysis window as a function of the implicit limit for the three block of stimuli. Lines correspond to the best linear fits to the data. (**d**) Comparison between the behavioral constant error (mean ± SEM) and the time difference between the Gaussian functions of the peak time of the boundary neurons (blue) determined with the fixed analysis window as a function of the implicit limit for the three blocks of stimuli.

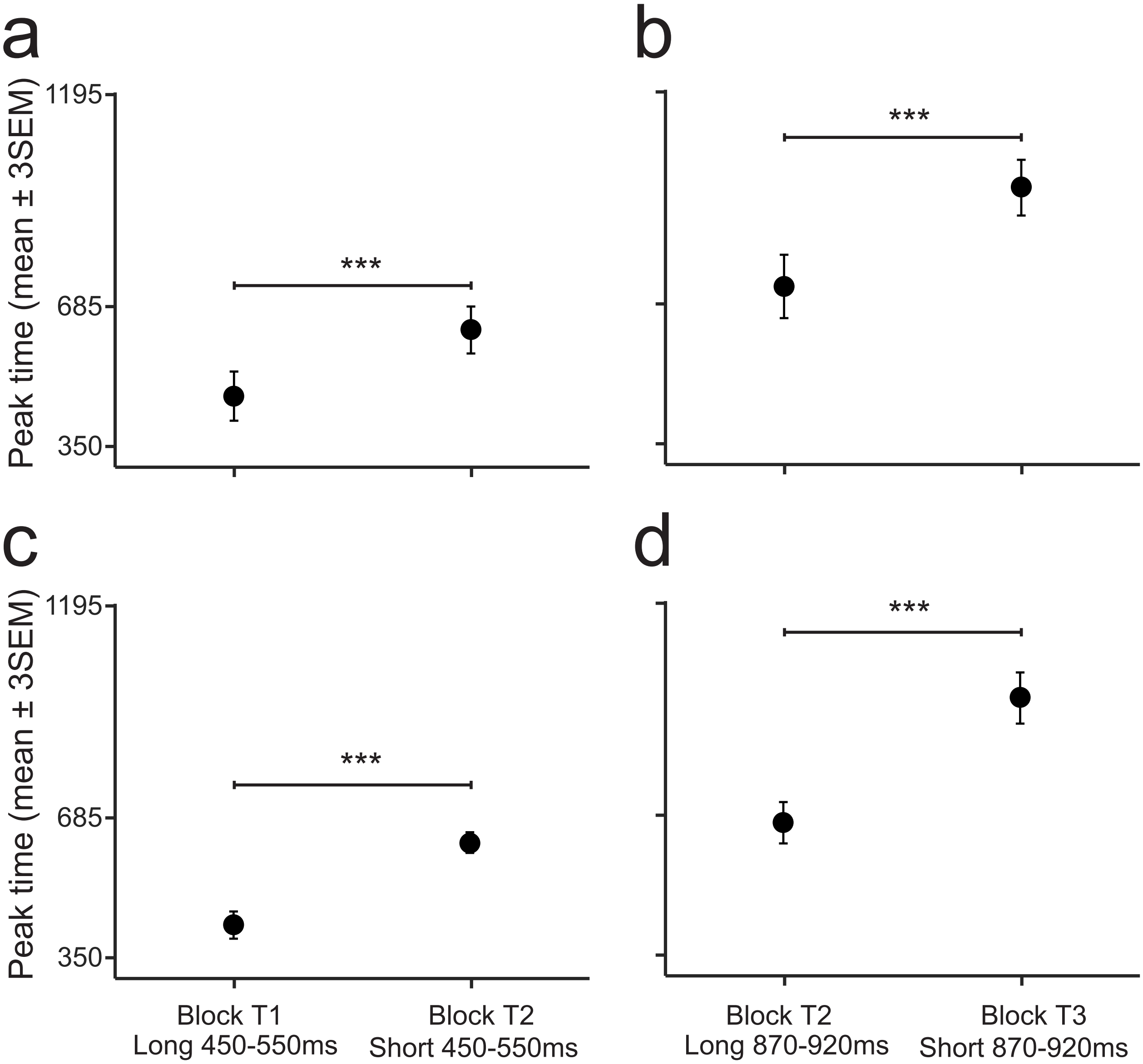

Supplementary Figure 6. (**a**) Times of peak activity (mean + 3SME) of boundary neurons in blocks T1 and T2 calculated using the same window size across blocks of intervals. Only cells with boundary activity in the two blocks were analyzed, comparing the peak times on the two test intervals with the same duration across blocks. The analysis window started 500 before the first stimulus and ended 500 ms after the second stimulus. Wilcoxon rank sum test, $p < 0.00001$, N = 49. (**b**) Same as in (a) for the test intervals of blocks T2 and T3. Wilcoxon rank sum test, $p < 0.00001$, N = 15. (**c**) Same as in (a) for the subpopulation of neurons encoding the boundary during T1 or T2. Wilcoxon rank sum test, $p < 0.00001$, $N_{T1} = 116$, $N_{T2} = 87$, (**d**) Same as in (b) for the subpopulation of neurons encoding the boundary during T2 or T3. Wilcoxon rank sum test, $p < 0.00001$, $N_{T2} = 87$, $N_{T3} = 32$.

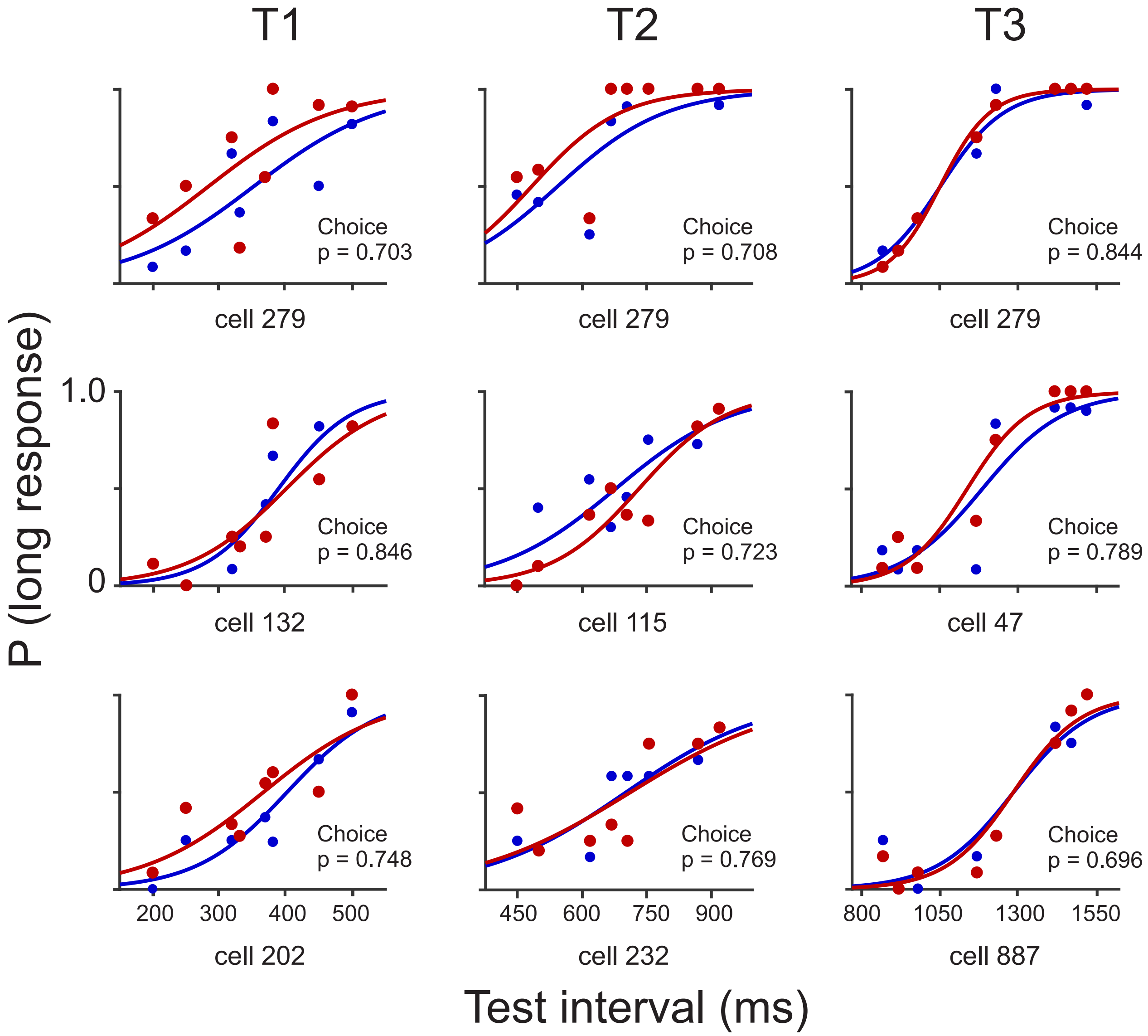

Supplementary Figure 7. Examples of boundary neurons whose activity is tightly associated with the categorical response of the monkeys. Neurometric functions (red), calculated from the decoded categorical choices of the monkey based on the τ of boundary neurons, and monkey's psychometric performance (blue) for the corresponding recording sessions. The mean boundary-choice probability for each neuron (see Methods) is indicated in the insets.

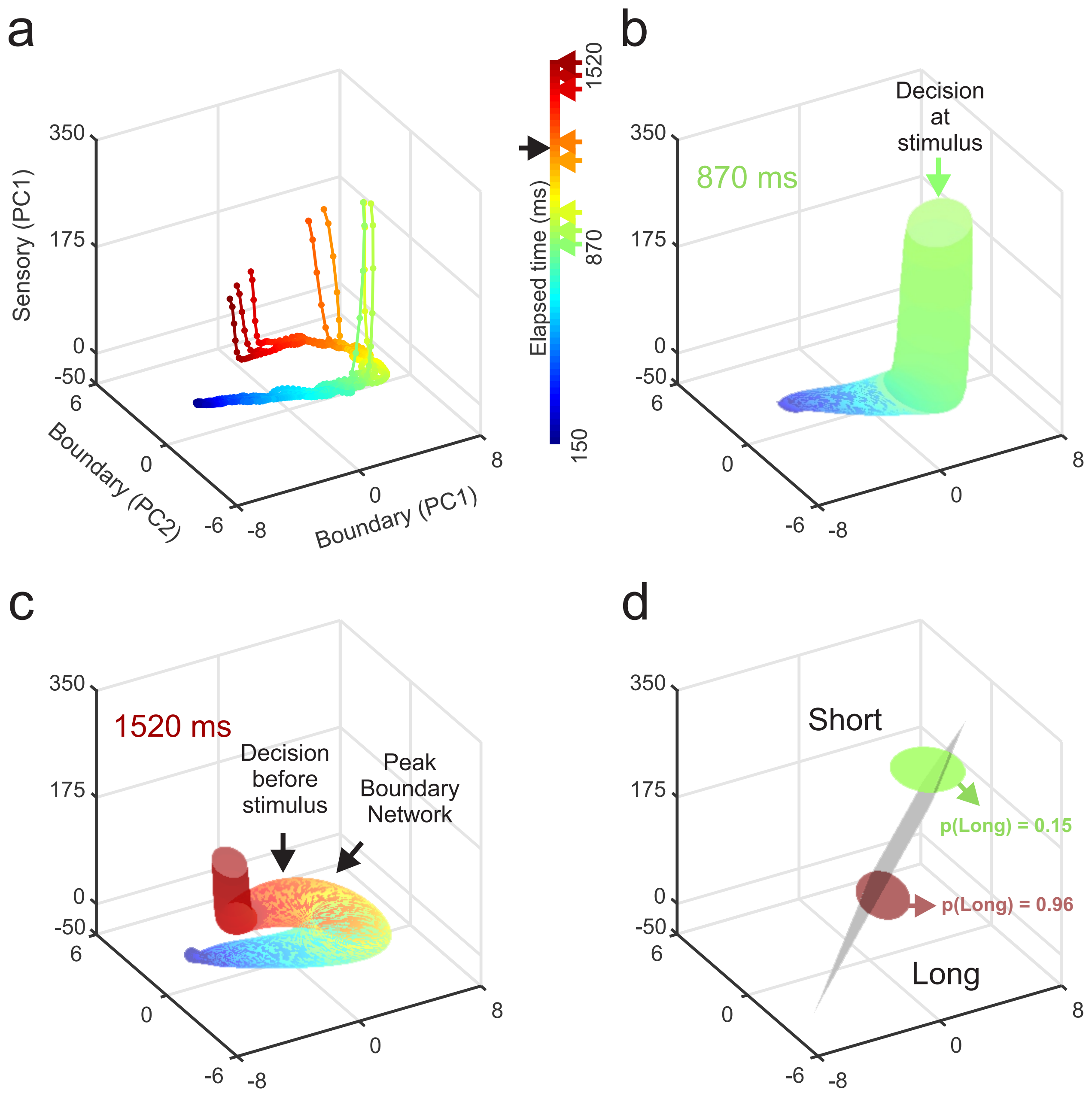

Supplementary Figure 8. PCA state trajectories of the activity of the sensory and boundary neural networks used to classify short and long durations. (**a**) PCA was carried out on the time-varying activity of cells in the sensory and boundary networks to produce population state trajectories in three dimensions. X and Y represent the PC1 and PC2 of the boundary network, respectively, while Z represents and PC1 of the sensory network. The network trajectories across simulations showed a curvilinear path for the PC1 and PC2 of the boundary network for all intervals. In contrast, the PC1 of the sensory network showed a sharp upward shift just after the second stimuli, which defines the end of the interval. The inset showed the color code of elapsed time, with the corresponding colored arrows for the eight test intervals of block 3. The black arrow corresponds to the threshold decision time from the optimization procedure for the linear classifier (see Methods). (**b**) Distribution of the network responses in PC space across 50 simulations over time (time bin 10 ms) for the shortest

interval of stimuli block 3 (870 ms). The diameter of each ellipse corresponds to the 50% of the trajectory covariance across simulations. The arrow indicates the time bin where the linear classifier found the best relation between the "networkmetric" and the psychometric performance of the monkeys for this interval. (**c**) The same as in (b) but for the largest interval of block 3. The peak time of the boundary network population is also indicated. (**d**) A linear classifier was used to find the plane in the PCA space that could divide short (green) and long (brown) intervals according to the psychometric performance of the monkey. The classification plane (gray plane) divided the space in short and long categorization responses. The proportion of simulations to the right and left of the plane was considered short and long, respectively. This classifier also found the decision time in the trajectory where the neurometric performance was closer to the monkeys' categorization behavior (corresponding to the arrows in (b and c)).

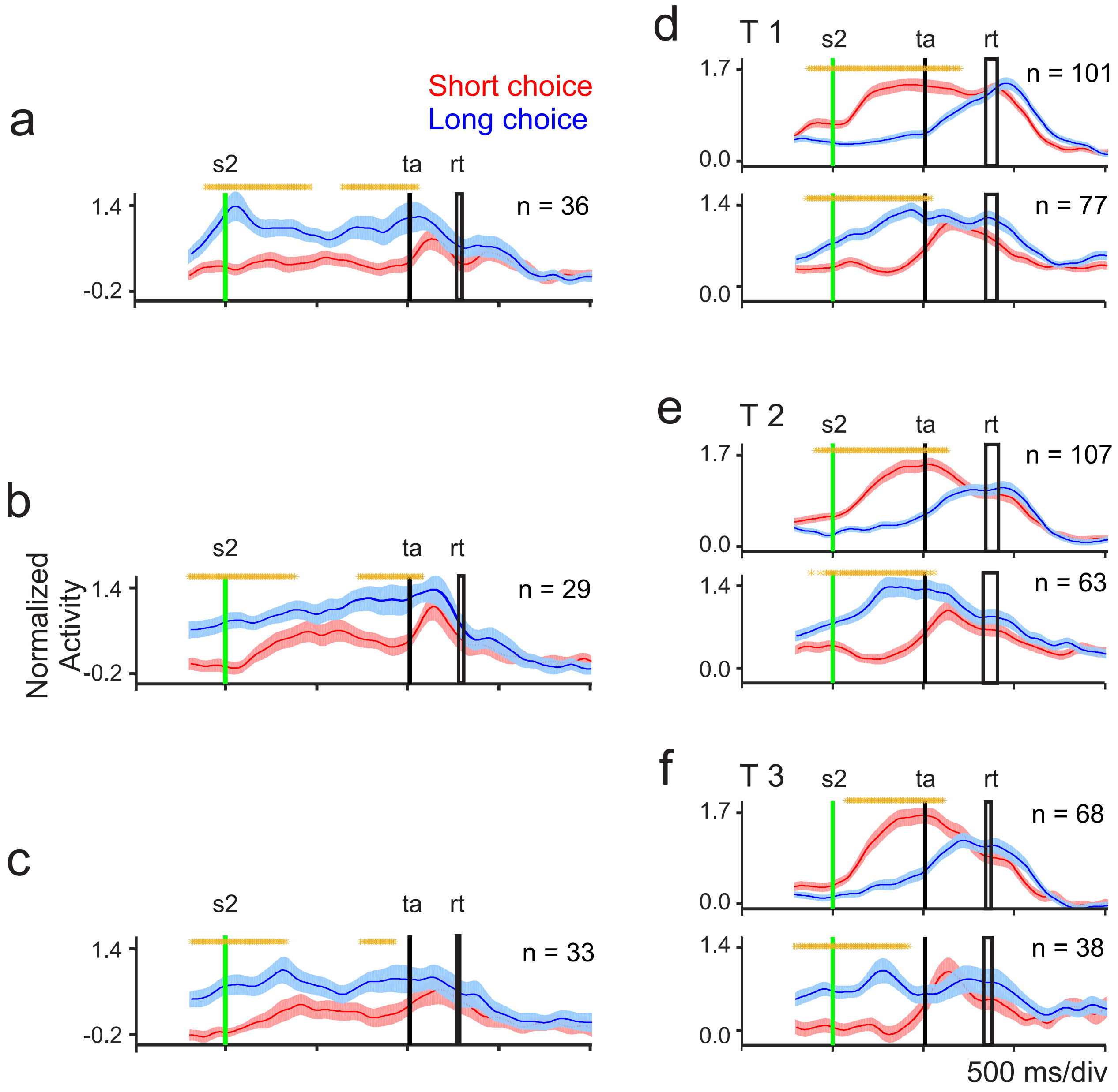

Supplementary Figure 9. Population activity of category-selective neurons during the delay epoch. (**a-c**) Population activity (mean ± SEM) of neurons of the monkey 1 with higher activity for the long choices across the three blocks of trials: (**a**) T1, (**b**) T2, and (**c**) T3. Orange asterisks indicate significant differences as determined by t-test in non-overlapping sliding windows (over the population SDF: short vs. long choices, 95% bootstrap confidence interval, 100 iterations). (**d-f**) Population activity (mean ± SEM) of neurons of the monkey 2 with larger activity for short (top panel) and long (bottom panel) categorical choices during the delay period, across the three blocks of trials: (**d**) T1, (**e**) T2, and (**f**) T3. Same notation as in (a-c). s2: second stimulus, ta: response targets, rt: + 1 SD of mean reaction time. In all cases the activity is aligned to the second stimulus.

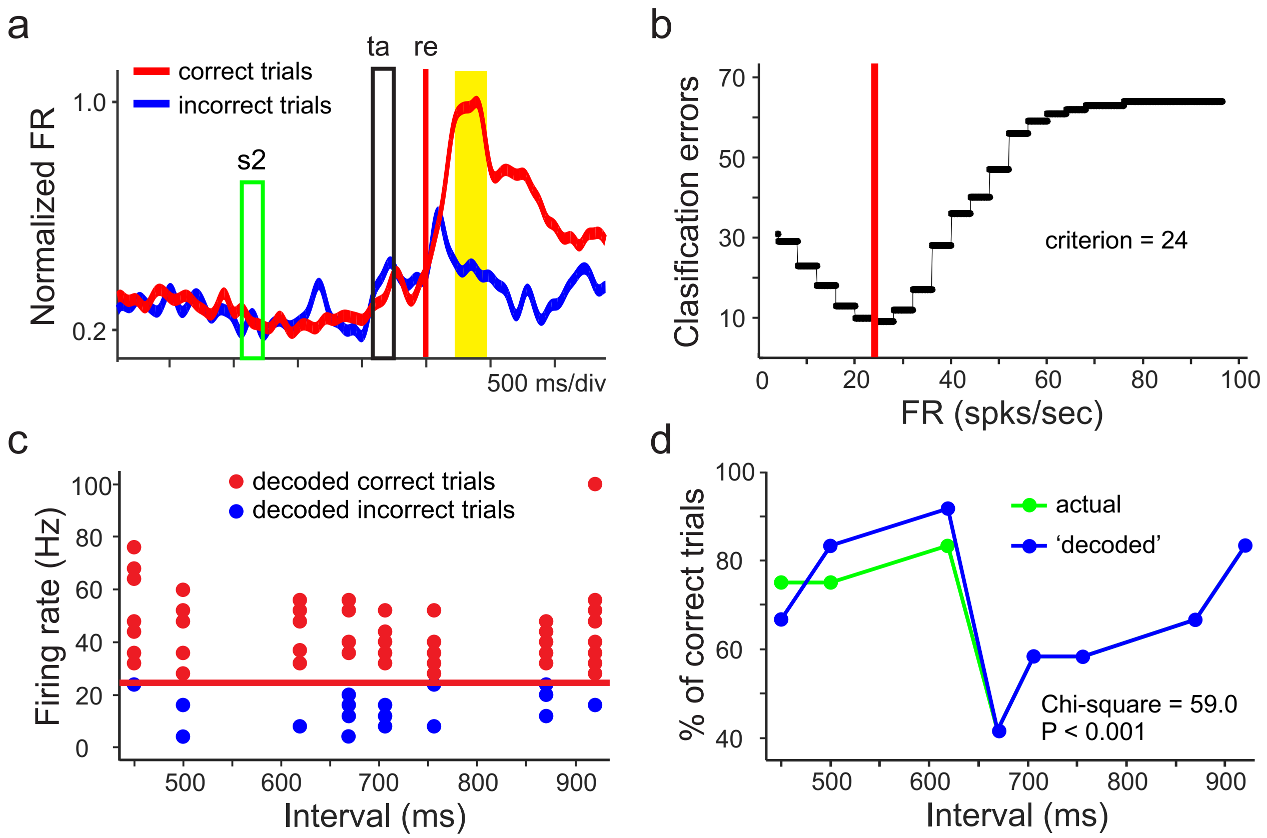

Supplementary Figure 10. Computing neuronal outcome-probability across all durations. (**a**). Mean normalized activity of a neuron showing larger activity for correct (red) than incorrect (blue) trials during the intertrial period for block T2. Vertical lines indicate, from left to right, the second stimulus (green), the response targets (black), and the end of the movement/time of reward (red). The activity is aligned to the reward time. (**b**). Number of errors in the correct/incorrect trial classification based on the activity of the neuron during the intertrial (yellow period in (a)), using a categorization cut criteria with a range from 0 to 75 Hz in steps of 1 Hz. The best limit criterion corresponds to 24 Hz, depicted as the red vertical line. (**c**). Discharge rate of the neuron during the same intertrial yellow period in (a), where trials above and below the criterion are considered correct (red) and incorrect (blue) trials, respectively. (**d**). Probability of correct outcome as a function of test interval for the actual behavioral outcome (green) and the neuronal outcome classification (blue, based on (c)). The chi-square test on the contingency table calculated between the behavioral outcome and the neural classification for the 96 trials was $x^2 = 59.0$ (P< 0.001).

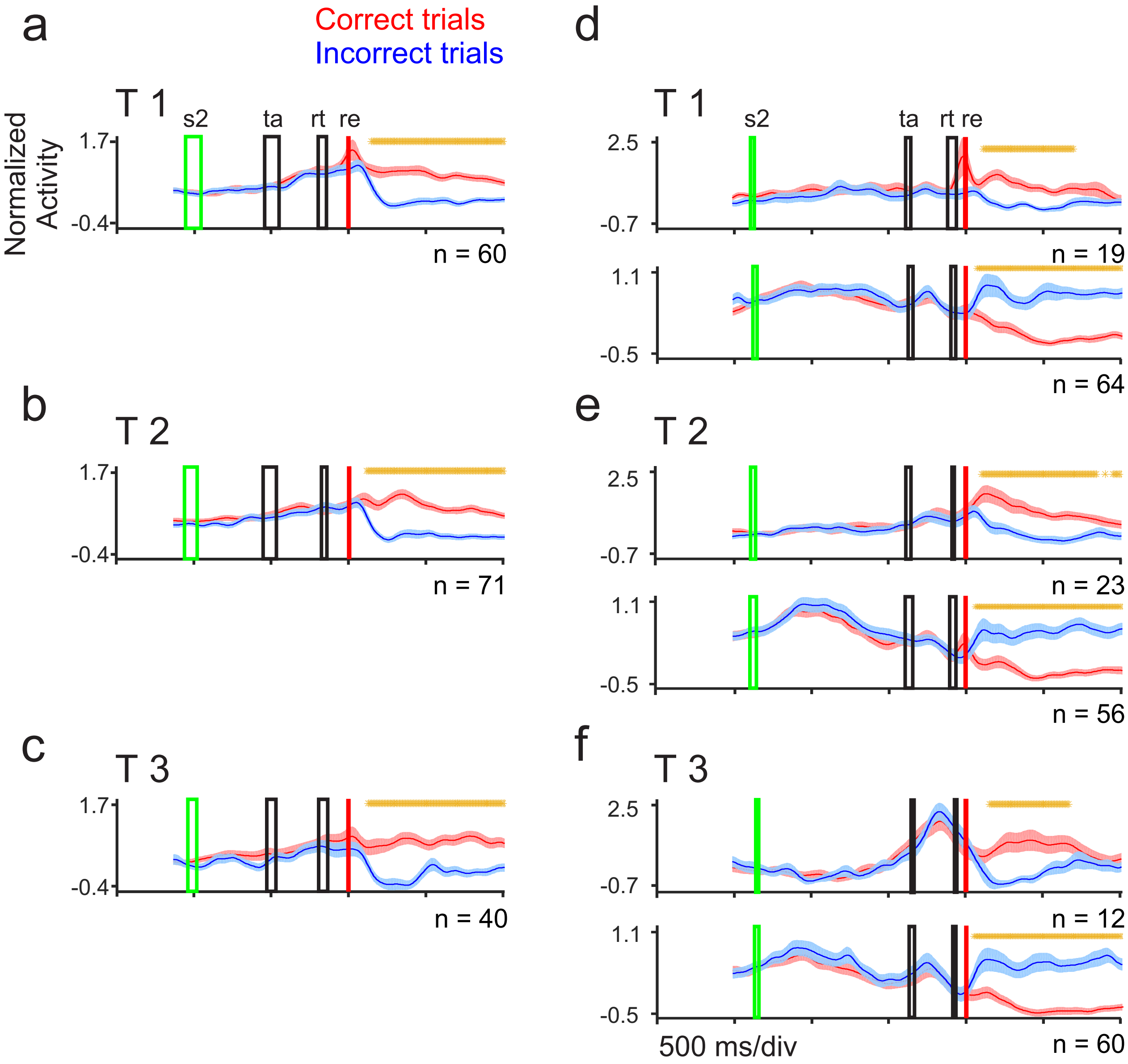

Supplementary Figure 11. Outcome neurons encode whether a trial was correct or incorrect during the intertrial period. (**a-c**) Normalized activity (mean Â± SEM) for the neurons of the monkey 2 that showed larger responses for correct (top panel) or incorrect trials (bottom panel) in (**a**) T1, (**b**) T2 and (**c**) T3. Orange asterisks indicate significant differences as determined by a t-test in non-overlapping sliding windows (over the population SDF: correct vs. incorrect trials, 95% bootstrap confidence interval, 100 iterations). s2, ta, and rt indicate + 1 SD of the mean time of first stimulus, targets, and reaction time, respectively. (**d-f**) Normalized activity (mean Â± SEM) of neurons of the monkey 1 for correct and incorrect trials with the same notation of (a-c). In all cases, the activity is aligned to the time of reward (re, red line).

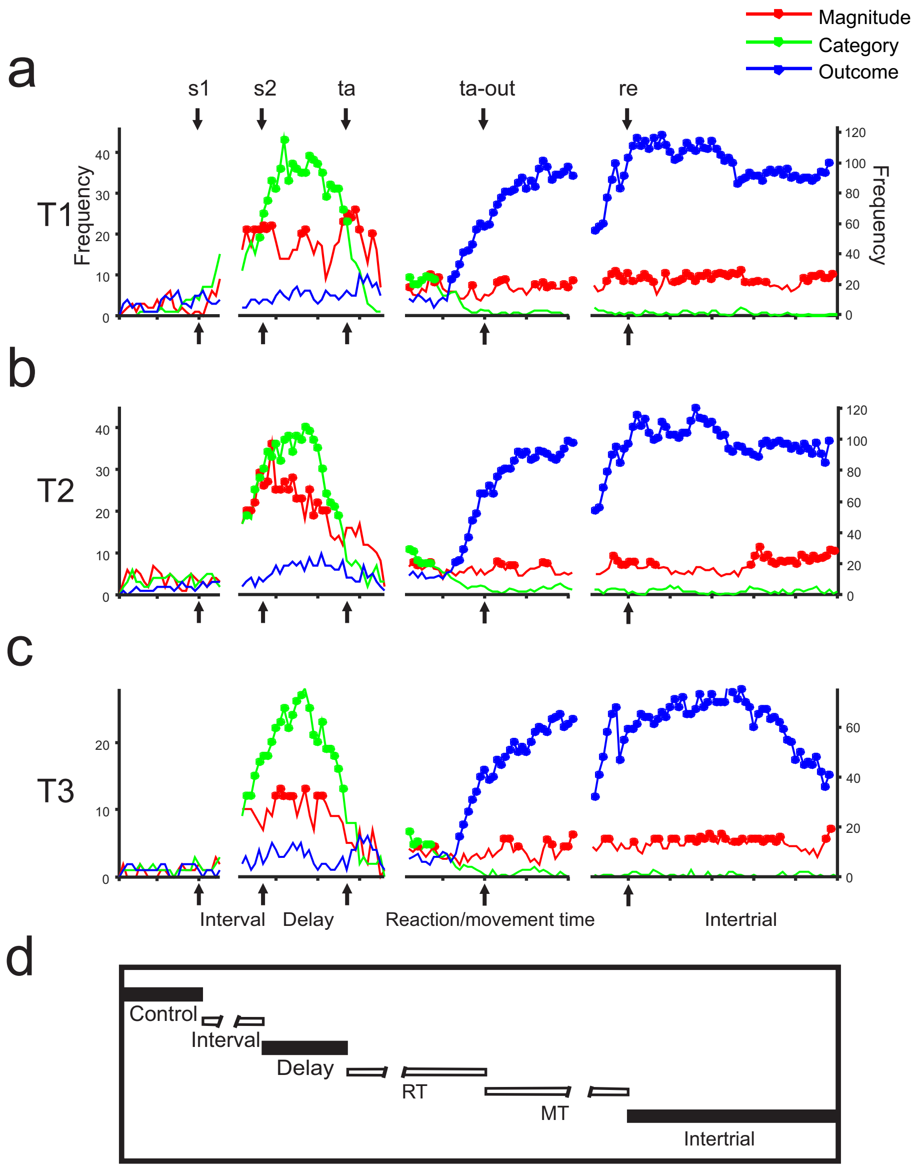

Supplementary Figure 12. Neural activity encoding stimulus duration, category and trial outcome emerged sequentially throughout the task. Frequency histograms for magnitude, category, and outcome related neurons as a function of the time throughout the categorization task for the (**a**) T1, (**b**) T2 and (**c**) T3 blocks of intervals. In this case, the magnitude-related cells are neurons whose overall discharge rate was modulated as a function of the interval duration according to the multiple linear regression model described in the methods. s1: first stimulus, s2: second stimulus, ta: targets, ta-out: cursor out of center circle, re: reward. Left axis indicates the frequency for the two panels to the left, right axis indicates the frequency for two panels to the right of the figure. Dots indicate time bins where the frequency of significant neurons for one of the three factors of the multiple regression (according to the permutations test) was above chance based on the total number of neurons recorded in the corresponding block of stimuli. (**d**) The temporal sequence of the different epochs of the task is shown for reference.

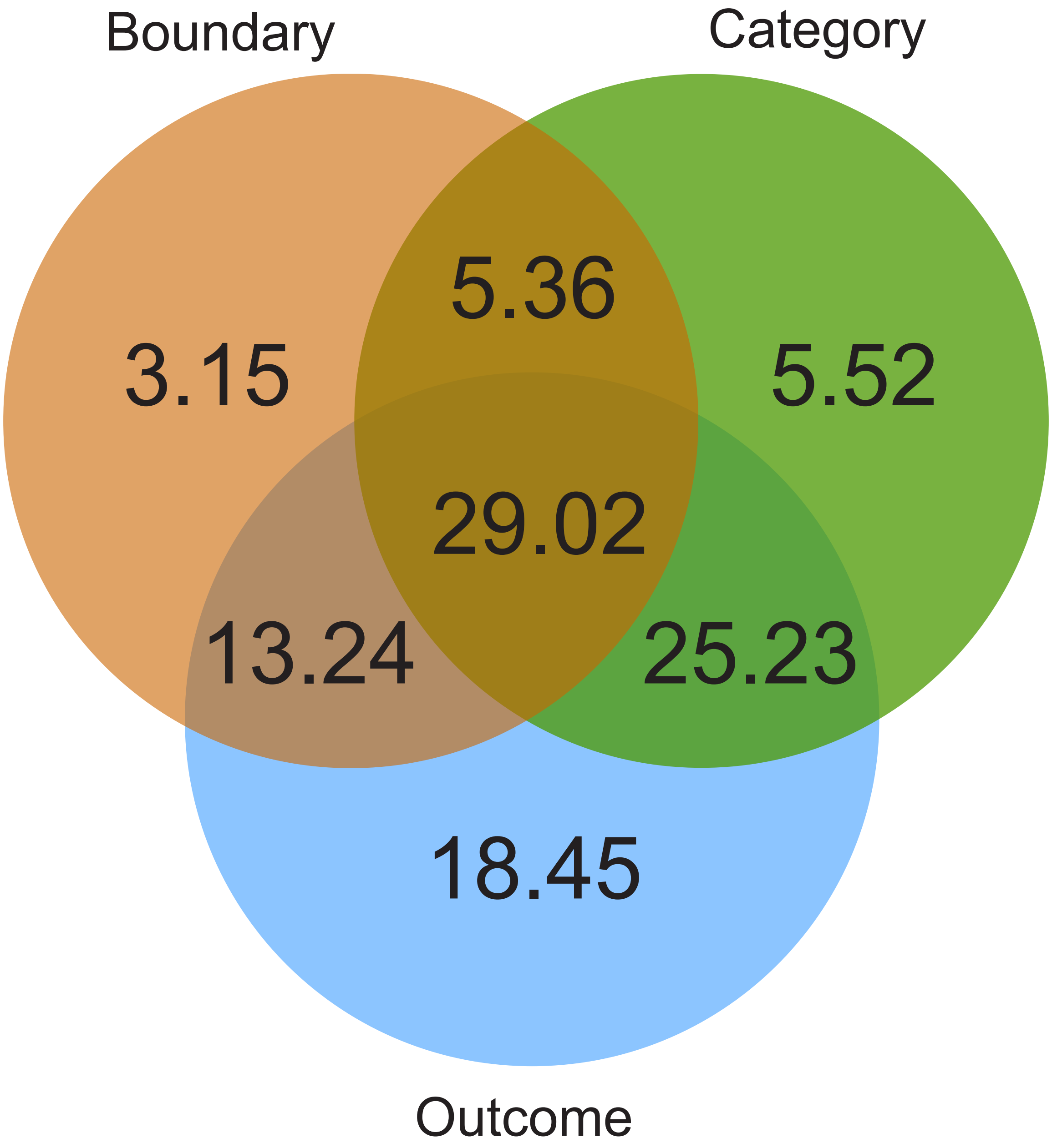

Supplementary Figure 13. Venn diagrams of the proportion of bisection, category and outcome selective populations for both monkeys. Note the large overlap between cell populations.

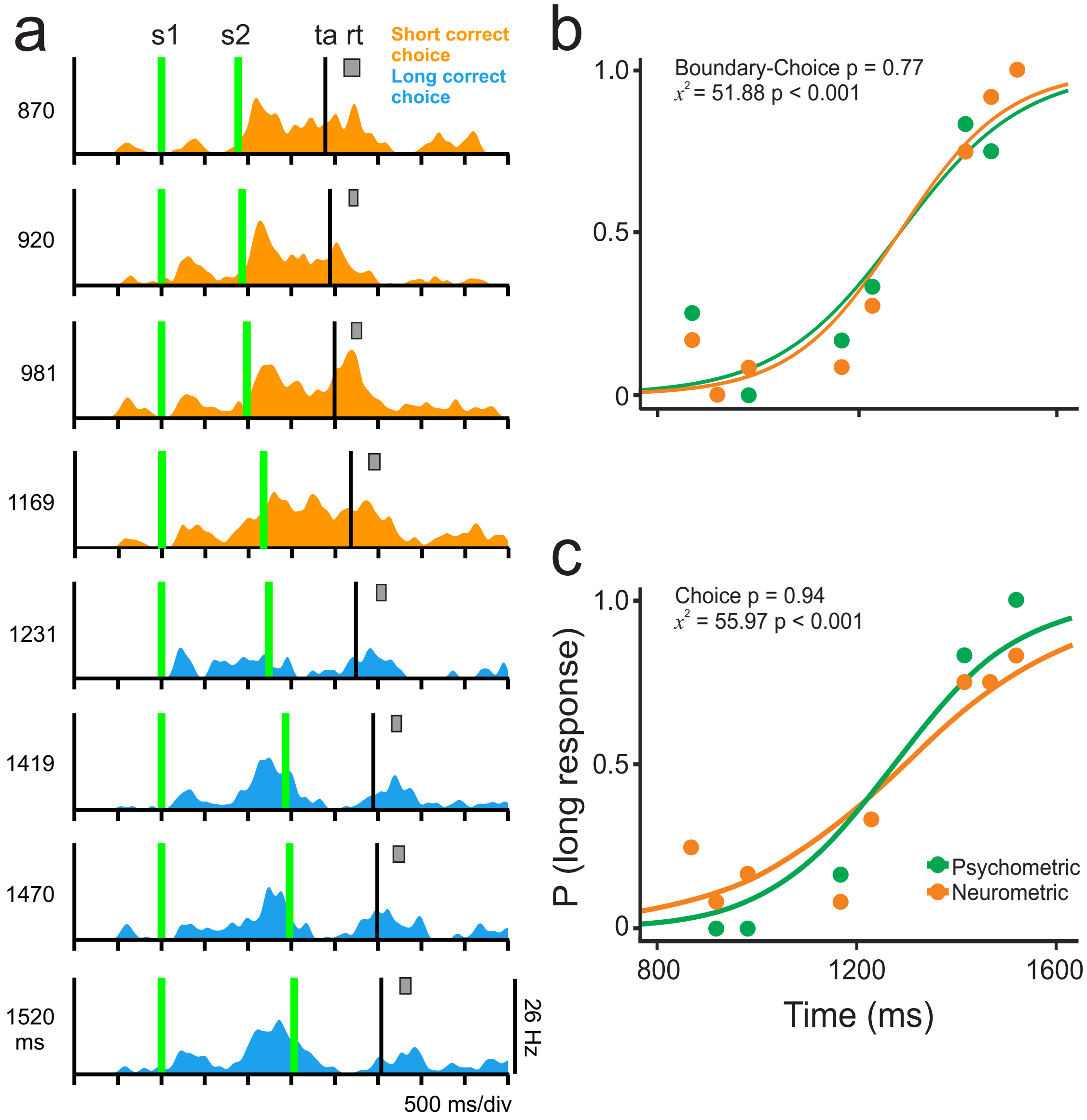

Supplementary Figure 14. Boundary neuron showing additional selectivity for category during the delay epoch. (**a**) Mean SDFs of a neuron for the eight test intervals of the T3 stimulus block. The peak activity occurs after the second stimulus for short intervals and before the second stimulus for long intervals. In addition, the activity during the delay epoch is higher for short choices. Abbreviations as in Fig. 2b. (**b**) Neurometric function (orange) of the neuron in (a) calculated from the decoded categorical choices of the monkey based on the τ, and monkey's psychometric performance (green) for the corresponding recording sessions. The Boundary-Choice Probability and the chi-square test on the contingency table calculated between the decoded and the observed monkey's choices across all 96 trials are indicated. (**c**) Psychometric performance (green) of the monkey during the recording of the neuron in (a), whose neurometric function is shown in orange. Neurometric function was calculated employing the mean firing rate during the delay epoch (s2 to ta period). The Choice Probability of this cell was close to 1 and the chi-square test on the contingency table calculated between the decoded and the observed monkey's choices across all 96 trials was significant ($x^2 = 55.97; p < 0.001$).
